# The Nanomechanical Properties of CLL Cells Are Linked to the Actin Cytoskeleton and Are a Potential Target of BTK Inhibitors

**DOI:** 10.1097/HS9.0000000000000931

**Published:** 2023-07-21

**Authors:** Marta Sampietro, Valeria Cassina, Domenico Salerno, Federica Barbaglio, Enrico Buglione, Claudia Adriana Marrano, Riccardo Campanile, Lydia Scarfò, Doreen Biedenweg, Bob Fregin, Moreno Zamai, Alfonsa Díaz Torres, Veronica Labrador Cantarero, Paolo Ghia, Oliver Otto, Francesco Mantegazza, Valeria R. Caiolfa, Cristina Scielzo

**Affiliations:** 1 School of Medicine and Surgery, BioNanoMedicine Center NANOMIB, Università di Milano-Bicocca, Vedano al Lambro, Italy; 2 Unit of Malignant B cells biology and 3D modelling, Division of Experimental Oncology, IRCCS Ospedale San Raffaele, Milan, Italy; 3 Unit of Microscopy and Dynamic Imaging, Centro Nacional de Investigaciones Cardiovasculares (CNIC), Madrid, Spain; 4 Unit B Cell Neoplasia, Division of Experimental Oncology, IRCCS Ospedale San Raffaele, Milan, Italy; 5 Università Vita-Salute San Raffaele, Milan, Italy; 6 Strategic Research Program on CLL, Division of Experimental Oncology, IRCCS Ospedale San Raffaele, Milan, Italy; 7 Klinik für Innere Medizin B, Universitätsmedizin Greifswald, Fleischmannstr, Germany; 8 Deutsches Zentrum für Herz-Kreislauf-Forschung e.V., Standort Greifswald, Universitätsmedizin Greifswald, Fleischmannstr, Germany; 9 Zentrum für Innovationskompetenz: Humorale Immunreaktionen bei kardiovaskulären Erkrankungen, Universität Greifswald, Fleischmannstr, Germany; 10 Institute of Physics, Universität Greifswald, Felix-Hausdorff-Strasse, Germany; 11 Experimental Imaging Center, IRCCS Ospedale San Raffaele, Milan, Italy

## Abstract

Chronic lymphocytic leukemia (CLL) is an incurable disease characterized by an intense trafficking of the leukemic cells between the peripheral blood and lymphoid tissues. It is known that the ability of lymphocytes to recirculate strongly depends on their capability to rapidly rearrange their cytoskeleton and adapt to external cues; however, little is known about the differences occurring between CLL and healthy B cells during these processes. To investigate this point, we applied a single-cell optical (super resolution microscopy) and nanomechanical approaches (atomic force microscopy, real-time deformability cytometry) to both CLL and healthy B lymphocytes and compared their behavior. We demonstrated that CLL cells have a specific actomyosin complex organization and altered mechanical properties in comparison to their healthy counterpart. To evaluate the clinical relevance of our findings, we treated the cells in vitro with the Bruton’s tyrosine kinase inhibitors and we found for the first time that the drug restores the CLL cells mechanical properties to a healthy phenotype and activates the actomyosin complex. We further validated these results in vivo on CLL cells isolated from patients undergoing ibrutinib treatment. Our results suggest that CLL cells’ mechanical properties are linked to their actin cytoskeleton organization and might be involved in novel mechanisms of drug resistance, thus becoming a new potential therapeutic target aiming at the normalization of the mechanical fingerprints of the leukemic cells.

## Graphical abstract

**Figure:**
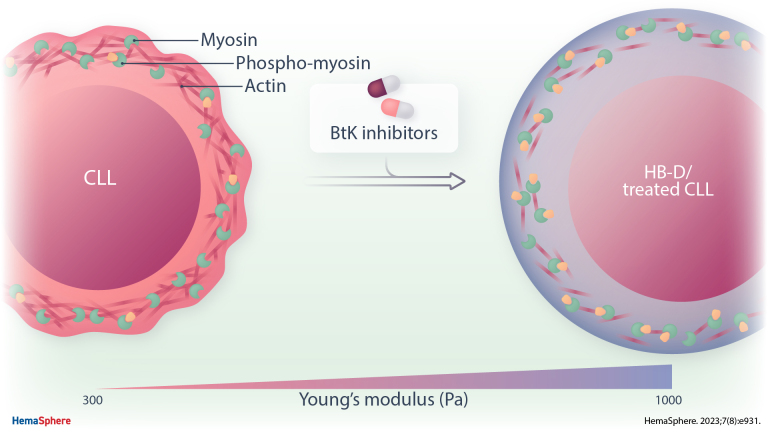

## INTRODUCTION

Chronic lymphocytic leukemia (CLL) is the most common leukemia in the Western world and it is characterized by progressive accumulation of mature monoclonal CD5^+^ B lymphocytes in the peripheral blood (PB), bone marrow (BM), and secondary lymphoid organs.^1^ The traffic of CLL cells^2^ between the PB and lymphoid organs is thought to be an active process involving a dynamic cytoskeletal remodelling^3^ that contributes to disease maintenance and progression, creating niches where CLL cells can survive and proliferate.^4^ These mechanisms represent a portion of a more complex scenario outlining a dynamic and heterogeneous disease,^5^ a complexity that may underlie the fact that CLL still remains incurable. Deeper understanding of CLL and the B-cell receptor (BCR) signaling pathway has resulted in the development of new therapeutic approaches that have remarkably improved patient outcomes.^6^ Among them, new target therapies (ie, kinase and BCL2 inhibitors)^6,7^ are progressively replacing chemoimmunotherapy. In particular, the Bruton’s tyrosine kinase (BTK) inhibitors^8^ such as ibrutinib,^9,10^ are an effective therapy leading to sustained responses, although patients may become resistant and relapse.^11^ Ibrutinib promotes CLL cells mobilization from the tissues to the PB^12^ where they lose the protective effect exerted by the microenvironment and eventually undergo apoptosis. However, what really occurs in the tissues, and which are the processes regulating CLL cells dynamics and resistance to the therapy remains to be elucidated also due to methodological limitations.^4^ An important aspect currently unexplored in CLL is how leukemic cells are able to adapt and remodel themselves and their microenvironments, once exposed to physical forces (ie, shear stress and compression).^13–16^ In particular, cells are able to sense^17,18^ these forces through mechanoreceptors and respond to them by exerting reciprocal cytoskeletal (ie, actomyosin complex) dependent generated forces, through a process termed mechanoreciprocity.^19^ Loss of mechanoreciprocity has shown to promote cancer progression in solid tumors^20,21^; however, just a few reports studied how the cytoskeleton and cell-intrinsic mechanical properties might affect hematological cancer development, progression, and response to therapies.^22,23^ In the present work, we combined different techniques to gather information at the single-cell level on cytoskeleton organization and the mechanical properties of primary B lymphocytes isolated, from patients with CLL and healthy donors (HD-B). We compared the actomyosin complex architecture by super resolution microscopy (stimulated emission depletion microscopy [STED]^24^), cellular swelling by osmotic shock,^25^ cell elasticity by atomic force microscopy in force spectroscopy mode (AFM-FS),^26–28^ and real-time deformability cytometry (RT-DC),^29,30^ identifying a significant different mechanical behavior of CLL cells in comparison to HD-B.

Furthermore, AFM-FS was employed to investigate the effect of ibrutinib on the mechanical properties of B cells. Importantly, we observed that in vitro and in vivo ibrutinib administration restores the mechanical properties of CLL cells toward the phenotype of HD-B lymphocytes until the onset of drug resistance and activates the actomyosin complex. All these data suggest that the mechanical fingerprint of CLL cells is linked to the actin cytoskeleton and is strongly associated with their malignant behavior and that the benefits of ibrutinib administration may also include the restoration of cell stiffness to physiological levels.

## MATERIALS AND METHODS

### Patients

Patients with CLL were diagnosed according to the updated National Cancer Institute Working Group guidelines.^31^ PB samples were obtained after informed consent from patients who were either (1) untreated or off treatment for at least 6 months or (2) under ibrutinib treatment. The study was approved by the Ospedale San Raffaele ethics committee under the protocol VIVI-CLL entitled: “In vivo and in vitro characterization on CLL.” Clinical and biological characteristics of CLL patients used for the experiments are reported in the Supplemental material (Table 1).

### Stimulated emission depletion microscopy

We used a gated STED-3X-WLL SP8 microscope (Leica Microsystems, Wetzlar, Germany) and a HC Pl Apo CS2 100×/1.40 oil objective for all experiments. The microscope was equipped with 592-nm and 660-nm depletion lasers, and the excitation was provided by a pulsed white laser. The acquisition software was LAS X 3.5.6.21594 (Leica Microsystems, Wetzlar, Germany). Cells were stained with anti-myosin-Alexa-532 Ab and/or anti-actin-Alexa-568 Ab. STED images were acquired under X,Y depletion at 660 nm, deconvolved (Huygens software, Scientific Volume Imaging BV, Hilversum, The Netherlands) and analyzed by ImageJ/Fiji software (Image.net).^32^ For details see Supplemental methods section and previous protocol optimization.^24^

### Swelling experiment

Cells were seeded on polyornithine (1:10) precoated µ-Slide 8 Well (Ibidi, Gräfelfing, Germany) in 100 µL of PBS to maintain a low level of soluble in the culture. Time-lapse bright-field sequences were recorded using a spinning disk confocal-base Zeiss microscope (Zeiss, Cell Observer SD, Oberkochen, Germany). In some cases, we stained the nuclear chromatin with Hoechst 33342 solution, 1:2000 for <10 minutes and washed the sample before microscopy. Time-lapse sequences were acquired for 30 minutes at a rate of 1 frame/s. After the first 50 seconds, we injected 450 µL of MilliQ water in the medium using a submillimeter tube (500 µm in diameter). Cells were immediately swelling. Adaptive focus was used for following the volume increase. Time-lapse image sequences were segmented using an ad hoc software developed in MATLAB software (MathWorks inc, Natick, MA). The starting point is the 16-bit tiff images generated from the microscope system which, following a procedure based on the H-Maxima transformation algorithm, are segmented to identify the area of the different cells. The cell radius is considered as half of the major axis of each individual area.

### Atomic force microscopy in force spectroscopy mode

For AFM-FS measurement, primary cells were plated on adapted 34 mm petri dish (TPP, 93040, Trasadingen, Switzerland) coated o/n with poly-L-ornithine solution 0.01% (Merck, Darmstadt, Germany) in Roswell Park Memorial Institute medium supplemented with 10% fetal bovine serum for 2 hours in standard incubator with a concentration of 2 × 10^5^ cells/plate. After careful removal of medium, cells were washed twice with PBS. Measures were carried out in PBS supplemented with Ca^2+^ and Mg^2+^ (Thermo Fisher Scientific, Waltham, MA). AFM-FS measurements were performed with a Nanowizard II (JPK Instruments, Berlin, Germany) equipped with a square-based pyramid probe (MLCT-BIO, cantilever E, 0.1 N/m nominal spring constant).^33–35^ All measures were carried out at room temperature in PBS Ca^2+^ Mg^2+^. The calibration of each cantilever spring constant was performed by thermal noise method^36,37^ both in air and in PBS just before the measurements on every petri dish. Force-indentation curves were acquired with a maximum applied force of 1 nN, a 4 μm ramp length and a constant speed of 2 μm/s on a grid of 1 × 1 μm^2^ with 4 × 4 points. The force-distance curves were corrected for the bending of the cantilever to obtain the force-indentation curves. The evaluation of cellular elastic properties, described quantitatively through the Young’s Modulus (YM), was obtained by force-indentation curves analysis with the Hertz-Sneddon model, taking into account the shape of the tip^38,39^ (see Supplemental Digital Content). Each force-indentation curve was fitted by JPK data processing software (JPK Instruments, Berlin, Germany) up to about 500 nm of indentation depth. To prevent significant changes in morphology or viability of living cells, each petri dish was measured within 2 hours. After measurements, a cell count with Trypan Blue (Merck, C8273, Darmstadt, Germany) was performed and compared with a control petri dish kept in the incubator in the standard seeding medium. No significant difference in the death count was detected.

### Real-time deformability cytometry

Cell mechanical measurements have been carried out using an AcCellerator system (Zellmechanik Dresden, Germany) with a fluorescence module. Suspended cells were driven through a microfluidic chip with a 300-µm long constriction of 20 × 20 µm^2^ cross-section where they were deformed by shear and normal stresses.^29,30^ Frozen samples from both patients and healthy donors were centrifuged for 5 minutes at 1500 rpm and resuspended in CellCarrierB (PBS^-/-^ without Ca^2+^/Mg^2+^ and supplemented with 0.6% [w/v] methyl cellulose) to a final concentration of 5 × 10^5^ cell/mL. Measurements were performed at a total constant flow rate of 0.08 μL/s. A total of about 10,000 cells per sample were captured. Analysis of cell shape was made with ShapeOut software version 0.9.6 (Zellmechanik Dresden, Germany) using an area ratio of 1.05 (ratio between raw area and area within contour) to ensure that cell contour represented the cell periphery.

### Drug treatment for AMF-FS measurements

For treatment for AFM-FS measurement, Cytochalasin D (Sigma, C8273, Albuquerque, New Mexico) was added directly to PBS, incubated for 10 minutes, and then maintained in the plate during the measurement. Similar protocol was used for treatment with IgM (Southern biotech, 9023-01, Birmingham); 10 µL/mL of IgM was added directly to PBS and maintained in the medium during all measurements. The experiment lasted no longer than 2 hours to avoid saturation of the BCR signal. Ibrutinib (Selleckchem, S2680, Planegg, Germany) and acalabrutinib (Selleckchem, S8116, Planegg, Germany) were administered at concentrations of 1 μM or 10 μM^40^ to cells in suspension. After 4 hours incubation, the unbound drugs were washed out by centrifuging the cell suspension. Cells were then seeded on polyornithine precoated dishes (1:10) for 2 hours to obtain stable adhesion, as described above for AFM-FS experiments in the absence of the drug.

### Statistical analysis

Statistical analyses were performed with Graphpad (San Diego, CA) and Matlab (MathWorks inc, Natick, MA) software applying Mann-Whitney *U* test, considering statistically significant a value of *P* < 0.05 (*), and consequently *P* < 0.005 (**) and *P* < 0.0005 (***).

## RESULTS

### Nanoscale cytoskeleton architecture of CLL cells

**Figure 1.**
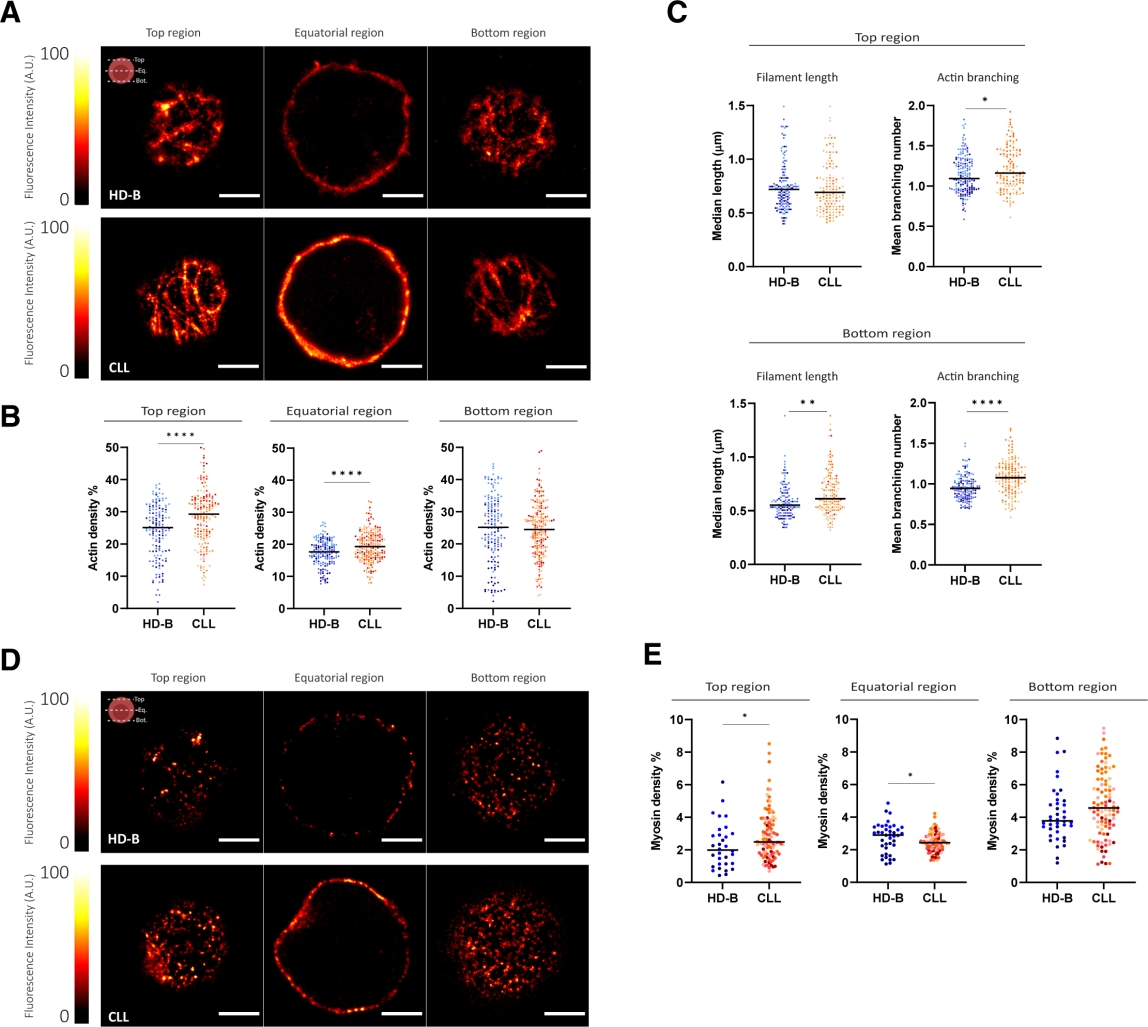
**Single-cell analysis by STED super resolution microscopy for actin.** (A) Representative images of anti-actin–Alexa-568 immunostained HD-B and CLLs cells acquired under identical experimental conditions at the three selected cellular regions. Scale bar = 2 µm. (B) Scatter plots of actin density expressed as percentage of the fluorescence intensity counts over the ROI total area intensity, obtained for the three explored cellular regions (see Materials and Methods). Cells derived from the same healthy donor (HD-B) or patient (CLL) are identified by color-code. Horizontal bars: median values. Top region: HD-B median 25.1%, and CLL median 29.3%; *P* < 0.0001. n = 147 HD-B cells from 3 donors and n = 181 CLL cells from 8 patients. Equatorial region: HD-B median 17.6%, and CLL median 19.3%, *P* < 0.0001. n = 158 HD-B cells from 3 donors and n = 213 CLL cells from 8 patients. Bottom region: HD-B median 24.7%, and CLL median 24.8%; *P* = 0.76, n = 159 HD-B cells from 3 donors and n = 156 CLL cells from 8 patients. (C) Actin morphology evaluated as length and branching of filaments at the top and bottom regions. In the scatter plots of actin filament length, we show the median filament length per cell. Top region: HD-B median 0.72 µm, and CLL median 0.69 µm, *P* = 0.34. n = 152 HD-B cells from 3 donors and n = 134 CLL cells from 5 patients. Bottom region: HD-B median 0.55 µm, and CLL median 0.61 µm, *P* = 0.002. n= 128 HD-B cells from 3 donors and n = 134 CLL cells from 5 patients. The filament branching plots illustrate the mean number of actin branching per filament and per cell. Top region: HD-B mean 1.10, and CLL median 1.16; *P* = 0.04. n = 164 HD-B cells from 3 donors and n = 139 CLL cells from 5 patients. Bottom region: HD-B mean 0.94, and CLL median 1.08; *P* < 0.0001. n = 142 HD-B cells from 3 donors and n = 146 CLL cells from 5 patients. (D) Representative STED images of anti-myosin-Alexa-532 immunostained HD-B and CLLs cells acquired under identical experimental conditions at the 3 selected optical regions. Scale bar = 2 µm. (E) Scatter plots of myosin density expressed as percentage of the fluorescence intensity counts over the ROI total area intensity as obtained for the three explored cellular regions (see Materials and Methods). Cells are color-coded according to each healthy donor (HD-B) or patient (CLL) from which they were obtained. Top region: HD-B median 2.0%, and CLL median 2.5%; *P* = 0.02; 32 HD-B cells from 2 donors and 101 CLL cells from 6 patients. Equatorial region: HD-B median 2.9%, and CLL median 2.4%; *P* = 0.03; 40 HD-B cells from 2 donors and 98 CLL cells from 6 patients. Bottom region: HD-B median 3.78%, and CLL median 4.57%; *P* = 0.06; 40 HD-B cells from 2 donors and 100 CLL cells from 6 patients. All graphical schemes were made with Biorender. CLL = chronic lymphocytic leukemia; HD = healthy donors; STED = stimulated emission depletion microscopy.

The nanoscale architecture of the actin cytoskeleton in primary healthy and leukemic B cells has never been explored so far. We applied single-cell STED super resolution microscopy optimized as previously published.^24^ We isolated primary B cells from PB of patients with CLL (n = 8; Table 1) and from HD-B donors (n = 3). Primary cells were plated on precoated polyornithine coverslips and stained for actin to visualize long and short actin filaments.^41^ For single-cell analysis, 3 optical sections were imaged at the top, equatorial, and bottom regions (Figure 1A), to compare, under identical acquisition conditions, actin density, filament length and branching in representative cell areas. Quantification was pursued by single optical section—single-cell analysis (Suppl. Figure S1). Actin filaments in CLL and HD-B cells are organized in a compact meshwork-like structure (Figure 1A). At the top regions, actin density in CLL cells was higher than in HD-B cells (*P* < 0.0001), while we did not observe significant differences at the bottom regions (Figure 1B). At the equatorial regions, actin appeared more packed and intense in CLL cells, depicting a thicker area in the cytosol (Suppl. Figure S2), and resulting in a higher density as compared with HD-B cells (*P* < 0.0001; Figure 1B). Next, we analyzed 2 structural elements of the actin-meshwork, filament length, and number of branching (Figure 1C; Suppl. Figure S3). It is known that filaments portions longer than 300 nm readily buckle under compressive forces involving the actomyosin complex,^42^ for this reason, we limited our analysis to the cell top and bottom regions where single filaments were detectable and we considered only filaments longer than 260 nm, also consistent with our experimental resolution (Suppl. Figure S4). Despite similar actin densities, significant differences were found at the bottom regions (Figure 1C; bottom panel), where CLL cells showed longer (*P* = 0.002) and more branched filaments (*P* < 0.0001) than normal HD-B cells. At the top regions where actin density was higher in CLL cells (Figure 1B), we observed only a moderate increase of branching (*P* = 0.04) but no longer filaments (Figure 1C; upper panel). The diversity of the actin organization captured by our analysis on 3 optical sections was confirmed with whole-cell reconstructions as shown in the representative 3D-STED stack (Suppl. Videos S1 and S2). To evaluate the contractile force generators in the cellular cortex,^43,44^ we further determined the local density of the nonmuscular myosin IIA, known motor and cross-linker protein (Suppl. Figure S1). HD-B (n = 2) and CLL (n = 6) cells were stained for myosin and STED images were acquired at the top, equatorial, and bottom regions of single cells (Figure 1D). In contrast to actin filaments, myosin shows punctuate and diffuse patterns. In CLL cells, myosin density at the top regions was significantly higher than at the bottom regions (Figure 1E; top region *P* value = 0.02; bottom region *P* value = 0.06). At the equatorial regions, a significant increase of myosin in HD-B compared with CLL cells regions was observed (*P* = 0.03). We performed an in-silico analysis (Blueprint Consortium) on the expression levels of the myosin regulatory light chain 2 *(MLC2*) and myosin heavy chain, nonmuscle IIa (*MYH9*), comparing primary CLLs and HD-Bs. In-silico analysis confirmed that CLL cells overexpress these genes, in line with our STED results (Suppl. Figure S5), and consistently suggesting an altered cytoskeleton organization in CLL cells.

**Table 1:** Clinical and Biological Parameters of the Patients With CLL Used in the Experiments (n = 46)

Pt number	Rai	Binet	CD38 Result	IGHV Homology	IGHV Homology Outcome	FISH	Clinical course	Application
1	1	A	NA	NA	NA	Not done	Stable	AFM
2	0	A	12.3	100	uCLL	del13q14.3 (90.6%, homozygous 27.8%, heterozygous 62.8%)	Progressive	AFM (ibrutinb), RT-DC
3	0	A	12.4	94.04	mCLL	del13q14.3 (20%), del13q34 (16.5%)	Stable	AFM, RT-DC, STED (Ibrutinib)
4	1	A	0.68	95.49	mCLL	del13q14.3 (29%), del13q34 (17.4%)	Stable	AFM, RT-DC
5	0	A	5.2	92.28/96.18	mCLL	Not done	Stable	AFM
6	1	A	57.2	93.75	mCLL	Not done	Stable	AFM
7	0	A	19.1	95.14	mCLL	Not done	Stable	AFM, RT-DC
8	0	A	0.1	93.33	mCLL	Normal	Stable	AFM
9	0	A	0	96.26	mCLL	del13q14.3 (90%)	Stable	AFM
10	0	A	15.90	98.30	uCLL	normal	Progressive	AFM (ibrutinb)
11	1	A	2	95.53	mCLL	del17p13.1 (12%), +12 (80%)	Progressive	AFM (ibrutinb)
12	0	A	0	90.97	mCLL	Not done	Stable	AFM
13	0	A	0.1	100	uCLL	Normal	Stable	AFM, STED (Ibrutinib)
14	0	A	5	100	uCLL	Normal	Progressive	AFM, RT-DC
15	NA	NA	NA	91.67	mCLL	NA	Stable	AFM
16	1	NA	1	93.15	mCLL	del13q14.3 (92.8%)	Progressive	AFM
17	0	A	41.5	100	mCLL	del(11q) 84%, del(13q14.3) 97%	Progressive	AFM–BM
18	0	A	36.4	100	uCLL	del(13q14) 87.8%	Progressive	AFM–BM
19	0	A	3	88.9	mCLL	NA	Progressive	RT-DC
20	0	A	0.1	97.98	mCLL	Not done	Stable	RT-DC
21	0	A	2.42	92.36	mCLL	NA	Stable	RT-DC
22	0	A	4.6	100	uCLL	del(13q14) 8%	Progressive	RT-DC
23	NA	NA	NA	91.67	mCLL	del(17p) 12%	Progressive	RT-DC, STED
24	0	A	0.4	91.32	mCLL	NA	Stable	RT-DC, WB
25	0	A	0.58	93.4	mCLL	NA	Stable	STED, AFM (acalabrutinib)
26	0	A	0.4	93.75	mCLL	Normal	Stable	STED
27	0	A	0.8	96.53	mCLL	del(13q) 80%	Stable	STED
28	0	A	0.6	87.02	mCLL	NA	Stable	STED
29	0	A	0	88.54	mCLL	NA	Stable	Swelling
30	1	A	1.3	100	uCLL	NA	Stable	Swelling
31	0	A	NA	92.63	mCLL	NA	Stable	Swelling
32	1	A	0.8	96.2	mCLL	NA	Stable	Swelling
33	0	A	16.32	100	uCLL	del(17p)	Progressive	AFM (ibrutinb)
34	0	A	0.16	3.94375	mCLL	del13q14.3 (10%), del13q34 (8.4%), del11q22 (8.4%), del17p13 (11.2%)	Stable	AFM (IgM)
35	0	A	0.3	93.8	mCLL	del13q14.3 (75.5%), del13q34 (10%)	Stable	AFM (IgM)
36	0	A	0.1	92.71	mCLL	NA	Stable	WB
37	1	A	0	96.18	mCLL	del17p (11%), del13q14.3 (18%) e del13q34 (9%)	Progressive	WB
38	1	A	0.1	91.23	mCLL	NA	Stable	WB
39	2	B	9	100	uCLL	Trisomy 12 (65.5%)	Progressive	WB
40	1	A	0	95,09	mCLL	NA	Stable	WB, AFM (acalabrutinib)
41	0	A	18.7	99.66	uCLL	Trisomy 12 (75%)	Stable	STED (Ibrutinib)
42	1	A	NA	NA	NA	NA	Progressive	AFM-Lynph node
43	1	A	1.4	100	uCLL	Trisomy 12 (71.7%)	Progressive	AFM-Lynph node
44	NA	NA	NA	NA	NA	NA	Progressive	AFM-Lynph node
45	0	A	3.33	100	uCLL	del17p (91%) and del 13q14.3 (23.5%)	Progressive	AFM (acalabrutinib)
46	0	A	0.16	91.41	mCLL	Normal	Stable	STED

CLL = chronic lymphocytic leukemia; mCLL = mutated=good prognosis; NA = not available; uCLL = unmutated=good prognosis.

### The actomyosin complex is altered in CLL cells

**Figure 2.**
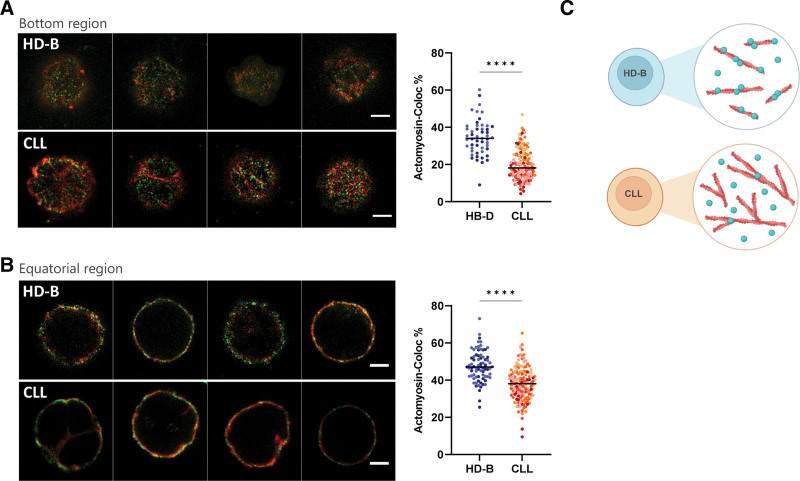
**Single-cell actomyosin colocalization by STED super resolution microscopy.** (A, left) Representative images of costained anti-myosin-Alexa-532 (green) and anti-actin-Alexa-568 (red) HD-B and CLL cells acquired under identical experimental conditions at the bottom region. Scale bar = 2 µm. (A, right) Quantification of the colocalization of myosin and actin by Manders’ coefficient expressed as percentage of colocalized actin pixels on myosin pixels. HD-B median 34.0%, and CLL median 18.2%; *P* < 0.0001; 51 HD-B cells from 2 donors and 150 CLL cells from 6 patients. (B; left) Representative images of costained anti-myosin-Alexa-532 (green) and anti-actin-Alexa-568 (red) representative HD-B and CLL cells acquired under identical experimental conditions at the equatorial region. Scale bar = 2 µm. (B; right) Quantification of colocalization of ActoMyosion with Manders’ coefficient expressed as percentage of colocalized myosin pixels on actin pixels. HD-B median 47.1% and CLL median 38.10%; *P* < 0.0001; 75 HD-B cells from 2 donors and 147 CLL cells from 6 patients. (C) Graphical representation made by Biorender of the actomyosin complex organization, where the blue dots represent myosin and the red fibrils represent actin. CLL = chronic lymphocytic leukemia; HD = healthy donors; STED = stimulated emission depletion microscopy.

Actin and myosin work as actomyosin complex to regulate cell contraction and cellular tension.^42–46^ We further analyzed the colocalization of actin and myosin in both primary HD-B and CLL cells (HD-B donors n = 2; CLL patients n = 6) to further study how the spatial arrangement of myosin motors at the cortex affects cortical tension and the possible involvement of this complex in CLL pathogenesis. Actin-myosin colocalization was analyzed in 2D-STED images of bottom and equatorial regions obtaining the percentage of colocalization of actin and myosin by Manders’ coefficient^47^ (Figure 2A and 2B), (Methods and Suppl. Figures S6 and S7). Actin density at the bottom regions did not change (Figure 1B), while we detected an increase of myosin density (Figure 1E) in CLL. Despite that, in those regions, actomyosin colocalization was found significantly reduced in CLL cells (Figure 2A; right panel; *P* < 0.0001). Moreover, although actin was denser at the cell equators (Figure 1A) in CLL, actin and myosin colocalization was higher in HD-B cells (Figure 2B; right panel; *P* < 0.0001). Noticeably, the loss of actomyosin colocalization was evident despite the intrapopulation heterogeneity (Figure 2). Considering myosin colocalization with actin as a sign of activation and contractility, our results suggest a remarkable cytoskeleton alteration in CLL, affecting the actomyosin complex (Figure 2C), possibly underlying a decreased cellular contractility and tension^42^ that may be responsible for altered mechanical responses.

### Different cytoskeletal architecture in CLL cells correlate with specific mechanical response

**Figure 3.**
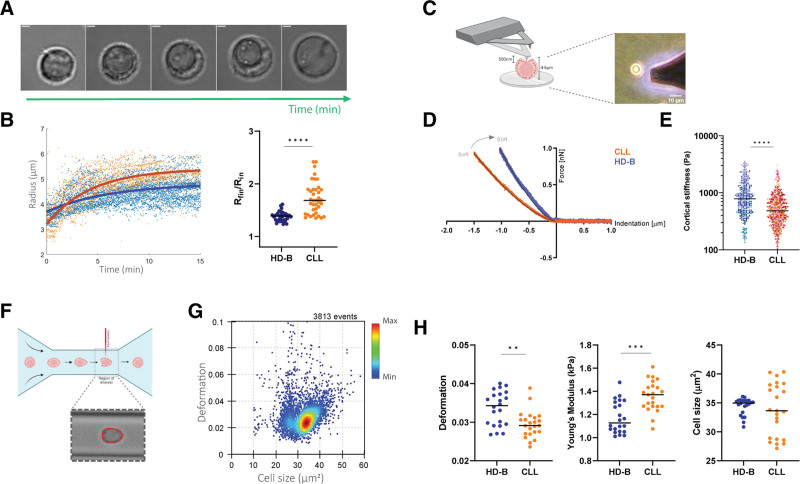
**Nanomechanical analysis of CLL and HD-B cells.** (A) Bright-field images of a representative 20 min time-lapse record of a single cell swelling as a consequence of osmotic shock. Scale bar = 5 µm. (B) Swelling kinetics of individual cells. (B; left) The continuous blue and red lines show global fitted curves on the total pool of HD-B and CLL cells respectively. The fit function is ([R_fin_-R_in_] × [1-exp[-t/τ]] + R_in_), where R_in_ and R_fin_ are the initial and final radius of the cell, t is the time and the τ is the swelling characteristic time. (B; right) Scatter plot of the swelling ratio (R_fin_/R_in_), HD-B median 1.39 and CLL median 1.68; *P* < 0.0001. 37 HD-B cells from 3 donors and CLL cells 39 from 4 patients. (C, left) Schematic representation (BioRender.com) of the AFM-FS basic principle, showing a cantilever pressing on a B cell at the cortical level. (C, right) The image shows a real snapshot of the AFM cantilever tip approaching a cell. Scale bar = 10 µm. (D) Representative force-indentation curves from an AFM experiment in the force spectroscopy mode for HD-B (blue dots) and CLL (orange dots) cells. Black lines are the fitting curves according to the Hertz-Sneddon model (see Methods for details). (E) Scatter plot of the cortical stiffness expressed as Young’s Modulus (Pa) of HD-B and CLL cells. HD-B median 785.1 Pa and CLL median 484.0 Pa; *P* < 0.0001; 345 HD-B cells from 9 donors and 765 CLL cells from 22 patients. Cells are color-coded according to each healthy donor (HD-B) or patient (CLL) from which they were obtained. (F) Biorender scheme of RT-DC, showing a cell passing through a microfluidic channel. Inset exemplifies bright-field image of a cell within region-of-interest, where deformation is obtained from red contour. (G) Representative scatter plot of cell deformation versus cell size (cross-sectional area) for n = 3813 HD-B cells analyzed by RT-DC. The color-code indicates a linear density scale. (H) Deformation, YM and cell size for HD-B from 7 donors and CLL from 6 patients. For deformation and YM each dot represents the median value of each individual experiment, with overall HD-B deformation median 0.034 and CLL median 0.029; *P* = 0.003 as well as overall HD-B Young’s Modulus median 1.13 kPa and CLL median 1.37 kPa; *P* < 0.0001. For cell size analysis each dot represents the mean cell size of each individual measurement with overall HD-B mean cell size 35.0 µm^2^ and CLL mean 33.6 µm^2^; *P* = 0.9. Measurements have been performed at a flow rate of 0.08 µL/s and statistical analysis has been done using linear mixed models. AFM-FS = atomic force microscopy in force spectroscopy; CLL = chronic lymphocytic leukemia; HD = healthy donors; RT-DC = real-time deformability cytometry; STED = stimulated emission depletion microscopy.

To further investigate cellular tension in response to external stimuli in HD-B and CLL cells, we performed a swelling experiment, measuring single-cell area in the presence of an osmotic pressure over time.^25,48–50^ As for STED imaging, cells were set in adhesion on precoated polyornithine in multi-well (HD-B donors n = 3; CLL patients n = 4). After water addition, cells swelled progressively, and their volume increased noticeably in the first 10 minutes (Suppl. Video S3; Figure 3A). The cellular radius over time was used to track hypoosmotic swelling (see Methods and Suppl. Figures S8 and S9) up to equilibrium. The cellular radius at equilibrium was calculated as the asymptotic fitting value (R_fin_) of an exponentially increasing function. The increase of the radius, expressed as R_fin_/R_in_ (where R_in_ represents the initial value of the radius) was undoubtedly more pronounced in CLL cells as obtained by the global fitting of the pooled kinetics (Figure 3B; *P* < 0.0001; Suppl. Figure S9). The same conclusion was reached by fitting each individual cell kinetics first, and then averaging the R_fin_/R_in_ values (Figure 3B). Thus, regardless the analytical method and despite swelling kinetics were characterized by heterogeneous distributions of characteristic times (τ) (Suppl. Figure S10). Results were in line with the different cytoskeleton organization in normal and patient-derived cells. The results suggest that a divergent response of the cells to tension and, ultimately, their altered mechanical properties might be a consequence of such a modified actomyosin complex in CLL cells.^44^ Considering that actin and myosin largely dominate the mechanical properties of cells and have an impact on cellular elasticity,^51,52^ we measured single-cell cortical stiffness by AFM-FS (Figure 3C).^53^ All measurements were performed with cells in adhesion and no changes in cell viability were observed (Suppl. Figure S11). We recorded force-indentation curves that describe the relationship between the applied force and the cell deformation (Figure 3D). We calculated the YM values by fitting the approaching force-indentation curve at <500 nm indentation depth, which can be referred to as cortical stiffness.^53^ The analysis of HD-B and CLL cells (from HD-B donors n = 9; CLL patients n = 22) indicated that CLL cells have a systematically lower cortical stiffness than HD-B cells (*P* < 0.0001; Figure 3E; Suppl. Figures S12 and S13). We further investigated whether there was a correlation between cortical stiffness and patients’ prognostic factors (Table 1). We examined the distributions of the cell cortical stiffness after segmenting patients according to the *IGHV* gene mutational status (*IGHV*<98%=mutated=good prognosis [mCLL, n = 16] and *IGHV*≥98%=unmutated=bad prognosis [uCLL, n = 5]).^54^ The results reported in Suppl. Figure S14A indicate a correlation between cell stiffness and *IGHV* mutational status, being uCLL cells significantly softer than the mCLL population (*P* = 0.04). In contrast, we did not observe any correlation with the disease progression (Suppl. Figure S14B).

Considering the intrinsic plasticity of lymphocytes and their ability to traffic and home in different anatomical compartments,^55–57^ we further assessed their mechanical properties under a different noncontact environmental constraint. We performed RT-DC measurements, which allows us to determine the elasticity of cells in suspension by shape analysis (Figure 3F).^29^ Each measurement summarizes the deformation and size of thousands of cells (Figure 3G). We then derived the median deformation for each experiment (Figure 3H), demonstrating that CLL cells deform less than HD-B (*P* = 0.003; HD-B donors n = 12; CLL patients n = 13). In parallel, a numerical model was used to calculate the elasticity value expressed as the YM (see Methods), where we noted a systematic softer behavior of HD-B cells compared with their leukemic counterparts (*P* = 0.0001). As for AFM-FS analysis, we segmented the patients according to the *IGHV* gene mutational status and diseased progression. We found that progressive CLL are significantly softer than stable CLL cases (*P* = 0.007; Suppl. Figure S15). Interestingly, we observed that CLL cells exhibit a wider intrinsically size heterogeneity with respect to HD-Bs; however, no relevant difference in the median area was noticed (Figure 3H).^58^ Moreover, we found a significant correlation between CLL cells area and the mutational status of the *IGHV* genes: uCLL (n = 3 patients) are smaller than mCLL (n = 8 patients) (*P* < 0.0001; Suppl. Figure S15), while YM value measured by AFM did not depend on cell size, showing a negligible Pearson correlation coefficient (data not shown). All together, these results confirm the differences between healthy and leukemic B cells and suggest that CLL mechanical adaptation properties are very dependent on the specific environmental cues as shown in suspension (circulation-like) for RT-DC and in adhesion (tissue-like) for AFM.

### CLL cells mechanical response can be tuned in vitro

**Figure 4.**
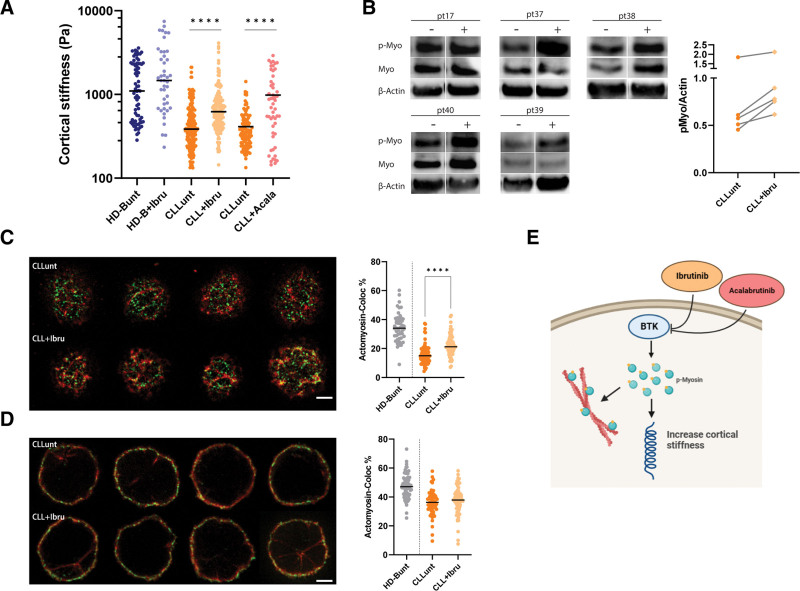
**Drug modulation of CLL cells mechano-response in vitro.** (A) Scatter plot of the cortical stiffness measured by AFM-FS of primary HD-B and CLL cells before (unt = untreated) and after drugs treatment (+Ibru= ibrutinib treatment; +Acala= acalabrutinib treatment). HD-Bunt median 1100 Pa, HD-B+Ibru median 1465 Pa; *P* = 0.1. 71 HD-Bunt cells and 44 HD-B+Ibru cells from 2 donors. CLLunt median 385 Pa and CLL+Ibru median 622 Pa; *P* < 0.0001; 211 CLLunt cells and 144 CLL+Ibru cells from 5 patients. CLLunt median 352.3 Pa, CLL+Acala 733.9 Pa; *P* < 0.0001. CLLunt 52 and CLL+Acala 102 from 3 patients. (B, left) WB analysis of cell lysates of CLL cells before (CLLunt) and after 4 h treatment with 10 μM Ibrutinib (CLL+Ibru) (n = 5). Bands represent respectively phospho myosin, total myosin, and β-actin. (B, right) Western Blot quantification of p-myosin increases upon treatment with ibrutinib in single patients. P-myosin increase was normalized as the ratio between p-myosin protein and actin (housekeeping gene). (C, left) Representative images of costained anti-myosin-Alexa-532 (green) and anti-actin-Alexa-568 (red) of CLLunt and CLL+Ibru upon treatment with 1 μM ibrutinib. Cells were acquired under identical experimental conditions at the bottom region. Scale bar = 2 µm. (C, right) Quantification of the colocalization of myosin and actin by Manders’ coefficient expressed as percentage of colocalized actin pixels on myosin pixels. HD-B as a reference in gray and CLLunt median 15% and CLL+Ibru 21%; *P* < 0.0001. 80 CLLunt and 69 CLL+Ibru from 3 patients. (D, left) Representative images of costained anti-myosin-Alexa-532 (green) and anti-actin-Alexa-568 (red) of CLLunt and CLL+Ibru upon treatment with 1 μM ibrutinib. Cells were acquired under identical experimental conditions at the equatorial region. Scale bar = 2 µm. (D, right) Quantification of the colocalization of myosin and actin by Manders’ coefficient expressed as percentage of colocalized actin pixels on myosin pixels. HD-B as a reference in gray and CLLunt median 36% and CLL+Ibru 38%; *P* = 0.3. 63CLLunt and 61 CLL+Ibru from 3 patients. (E) Schematic representation (BioRender.com) of the pathway involved in CLL cell’s mechanical properties modulation. Ibrutinib inhibition of the BTK induces an increase of myosin phosphorylation (blue and yellow spot), which is directly involved in the modulation of cellular mechanical properties. AFM-FS = atomic force microscopy in force spectroscopy; BTK = Bruton’s tyrosine kinase; CLL = chronic lymphocytic leukemia; HD = healthy donors; WB = Western Blot.

We further tested to which extent the mechanical properties of the cells could be modulated in vitro by specific stimuli. We verified that in none of the experiments, cellular viability was altered at the selected drug concentration (Suppl. Figure S16). First, we used mycotoxin cytochalasin D^59,60^ as a conventional stimulus known to prevent actin monomer polymerization,^61,62^ thus decreasing cell elasticity. Its effect was appreciable on HD-B cells, which displayed a significant decrease in cortical stiffness (*P* < 0.0001) (Suppl. Figure S17). On the contrary, the drug had no evident effect on CLL cells (Suppl. Figure S17), suggesting that their intrinsically low cortical stiffness could not be further modulated. These results prove that we can detect drug-mediated effects on lymphocytes’ cortical stiffness by AFM-FS and prompted us to further investigate this aspect using a clinically relevant drug. We analyzed whether the kinase inhibitor ibrutinib^9,10^ could have an impact on the mechanical properties of CLL cells. We incubated cells with ibrutinib for 4 hours at a final concentration of 1 μM and 10 μM^40^ and we did not observe any differences in the effect of the 2 concentrations (Suppl. Figure S18). Incubation with ibrutinib induced an increase of cortical stiffness in CLL cells (Figure 4A; *P* < 0.0001 and Suppl. Figure S19) to almost physiological level but did not significantly alter the response of HD-B cells (*P* = 0.1; HD-B donors n = 2; CLL patients n = 5). To confirm the direct involvement of BTK inhibition, we tested a more specific inhibitor namely acalabrutinib^8^ on 3 additional patients with CLL and we confirmed the increase in stiffness (*P* < 0.0001; Figure 4A). The possible involvement of the BCR receptor activation in this context was tested by stimulating CLL and HD-B cells with anti-IgM. We did not observe any changes of the cortical stiffness values (Suppl. Figure S20; HD-B *P* = 0.3; CLL *P* = 0.6) indicating that the BCR activation is not directly affecting B cell cellular stiffness. In addition, by western blot, we quantified the activation of p-myosin following ibrutinib treatment (Figure 4B) in order to evaluate the potential involvement of the actomyosin complex in the mechanism of action of ibrutinib in 5 CLL patients. We observed that ibrutinib can upregulate at variable levels myosin phosphorylation in vitro (Figure 4B, right panel; Suppl. Figure S21) in CLL cells, suggesting a drug modulation effect on the actomyosin complex. Therefore, we studied the cellular colocalization of the 2 proteins by 2-color 2D-STED microscopy before and after treatment with ibrutinib. We focused this analysis on the cell bottom where we did not see a significant difference between healthy B cell and CLL cells when we studied the 2 proteins individually (Figure 1). In this section, myosin and actin densities were unchanged as compared with the healthy counterparts but their colocalization decreases dramatically in CLL (Figure 2B). Interestingly, we observed a significant recovery of actin and myosin colocalization in these bottom cell sections (*P* < 0.0001) upon treatment with ibrutinib (Figure 4C). As comparison we also imaged the actomyosin complex at the cell equators, where we previously detected less difference in terms of myosin (Figure 1E), and we did not observe a significant change in the complex organization after ibrutinib treatment (Figure 4D). All together, these results suggest an effect of the drugs on both actomyosin complex and cortical stiffness (Figure 4E).

### CLL cells mechanical properties are reverted to normal phenotype by ibrutinib in vivo

**Figure 5.**
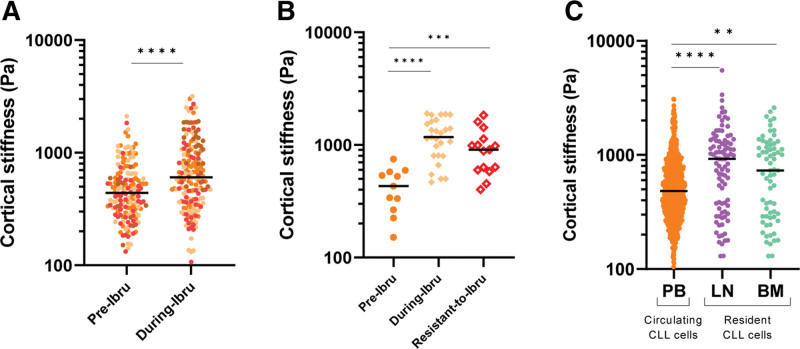
**Cortical stiffness measurement by AFM-FS of cells from patients with CLL under ibrutinib therapy.** (A) Scatter plot of the cortical stiffness of a pull of CLL cells from patients under treatment with ibrutinib at 4, 8, 9 and 22 weeks. Pretreatment (before starting the treatment) median 440 Pa, and during ibrutinib treatment median 600 Pa, *P* < 0.0001. 159 basal cells and 170 under ibrutinib treatment cells from 4 patients. Cells are color-coded according to each patient (CLL) from which they were obtained. (B) Scatter plot of the cortical stiffness of a patient at 3 time point during the course of the disease: Pretreatment (before starting the treatment), during ibrutinib treatment and resistant to ibrutinib (ones the patient relapse during the therapy). Basal median 430 Pa, Ibrutinib median 1175 Pa and resistant median 910 Pa. Basal vs Ibrutinib *P* < 0.0001, basal vs resistant *P* = 0.0002, and Ibrutinib vs resistant *P* = 0.06. 11 Basal cells, 22 cells under Ibrutinib treatment, and 15 cells resistant from 1 patient. (C) Scatter plot of the cortical stiffness of circulating CLL cells from PB and resident CLL cells from LNs and BM of patients. Circulating CLL median 484 Pa and resident CLL from LN median 926 Pa; *P* < 0.0001. CLL from BM median 731 Pa; *P* = 0.008. Circulating 765 CLL cells from 22 patients, LN 93 from 3 patients and BM 68 CLL cells from 2 patients. AFM-FS = atomic force microscopy in force spectroscopy; BM = bone marrow; CLL = chronic lymphocytic leukemia; LNs = lymph nodes; PB = peripheral blood.

In view of the above results, we asked whether the mechano-response observed in CLL cells in vitro might have a clinical relevance in patients under treatment. We evaluated by AFM-FS the cortical stiffness of single CLL cells isolated from PB of 4 patients before and during ibrutinib treatment (4, 8, 9, and 22 weeks, respectively). As shown in Figure 5A and Suppl. Figures S22 and S23, CLL cells isolated from patients under treatment showed a significant recovery of physiological cortical stiffness (*P* < 0.0001), mirroring what we observed in vitro, and confirming the rescue of circulating cells to a healthy phenotype during treatment. To prove that the mechanical modulation toward a healthy phenotype during treatment with ibrutinib is lost once the patients become resistant to therapy, we checked the cortical stiffness value of an ibrutinib-resistant patient. We measured, by AFM-FS, the cortical stiffness of single CLL cells isolated from PB of a patient at 3 different stages of the disease, CLL cells were collected: (1) at basal level before starting ibrutinib treatment; (2) during the first-line treatment while showing a clinical response to ibrutinib; (3) during treatment at the time of the onset of the resistance, before a second-line treatment. We confirmed an increase of the cortical stiffness during ibrutinib treatment, and interestingly we observed a trend of decreasing stiffness in CLL cells becoming resistant to ibrutinib, suggesting a reversal to the initial leukemic phenotype (Figure 5B). This observation prompted us to analyze CLL cells in the tissues where they could possibly behave differently based on the different environmental cues (CLL PB n = 19; CLL from Lymph node [LN] = 3; and BM n = 2). As shown in Figure 5C, tissue-resident CLL showed cortical stiffness values higher than those of circulating CLL cells (LN: *P* < 0.0001; BM: *P* = 0.008). The data suggest that the differences observed in the mechanical properties and mechano-response of CLLs as response to ibrutinib might also depend on the tissue localization of the lymphocytes and on their plastic properties.

## DISCUSSION

We here report the first study linking the actomyosin nanoarchitecture to the mechanical properties of primary CLL cells in comparison to healthy B cells at single-cell level.^22,63^ The driving hypothesis was that CLL cells frequently rearrange their cytoskeleton to favor continuous cell entry and egress from the tissue where they are exposed to the most disparate physical forces^15,64^ and they can modify their response to mechanical cues, and this potentially differs from HD-B cells.^65^ The implication of the cytoskeleton in the dynamic behavior of CLL cells was already observed in the past showing that CLL cells have impaired,^66^ and aberrant cytoskeleton rearrangement and activation.^56,67^ Moreover, the presence of the so-called smudge cells in the blood smear of the patients underlines a fragility of CLL cells that can be directly attributed to the cytoskeleton. The actin network links the extracellular environment with the inner of the cells and it is able to convert extracellular mechanical stimulation into a biological response, in fact the cytoskeleton is one of the major complexes involved in cellular mechanics.^68^ Mechanobiology has been a neglected aspect in CLL pathophysiology so far, although it can be very relevant especially for migrating cells as lymphocytes. This observation, together with the alterations that we found in the actomyosin complex by super resolution microscopy, prompted our investigation on the study of CLL mechanical phenotype by using complementary approaches. We here demonstrated that CLL cells have a cortical stiffness lower than HD-B cells. This result appears to disagree with the findings reported by Zheng et al,^22^ that obtained for CLL cells a YM value higher than for healthy cells. However, Zheng et al considered lymphocytes as a whole population, while we restricted our analysis only to B lymphocyte subpopulations. It is not unexpected to find specific mechanical properties for a cellular subgroup that might remain hidden in a mixed cohort. Keeping this in mind, we selected a heterogeneous cohort of CLL patients, both mutated and unmutated subgroups to assess whether our findings are generally true for the whole CLL cell population. Interestingly, although we have detected a clear difference between CLL and HD-B cells, we were able to observe that mCLL are stiffer than uCLL, thus more similar to their healthy counterparts. This suggests that cell softening could be part of the worsening of the disease. In line, by discriminating CLL samples based on the mutational status of the *IGHV* gene, by RT-DC we observed a correlation of the mutational status with the cellular size: mCLL were bigger than uCLL, confirming the less aggressive phenotype behavior more similar to that of HD-B cells. This observation also suggests that the size and the stiffness may reflect the functional differences in the type of antigenic interaction through the BCR; however, we could not find any significant variation in stiffness following BCR stimulation. RT-DC that allows the investigation of the mechanical properties of CLL cells in nonadhesive environments confirmed the noticeable differences between CLL and HD-B cells, highlighting the complementary readouts of our approaches. Along this line, swelling experiments showed that adhered CLL cells are more prone to modify their shape/deformation than HD-B cells, as for AFM-FS, proving that the environmental conditions (adhesion or suspension, tissue or circulation) can be determinant to define a specific mechano-response. This aspect is also clinically relevant considering that the primary action of ibrutinib (a first-line treatment for CLL) is to induce CLL cells mobilization from the tissues into the PB,^12^ with a poorly defined mechanism.

Previous reported AFM-FS analysis on 2 other types of leukemia cells^23^ showed that chemotherapy treatments renormalize the stiffness of the malignant cells. Accordingly, we hypothesized a possible involvement of ibrutinib in modulating the mechanical properties of CLL cells. Indeed, we were able to demonstrate the following in response to ibrutinib: (1) overall, HD-B cells exhibit an unaltered mechanical phenotype; (2) after exposure to drugs, leukemic cells recover the physiological range of cortical stiffness of HD-Bs; and (3) CLL cells show a partial recovery of actin and myosin colocalization and activation of the actomyosin complex. In addition, we proved the reproducibility of these observations also in vivo, in patients under ibrutinib treatment.

One of the possible mechanisms underlying our observations might be the ibrutinib inhibitory effect on lipid metabolism in CLL as proposed by recently published evidence.^69^ Moreover, Lei et al^53^ showed that depletion of membrane cholesterol induces an increase of stiffness of cancer cells. Therefore, ibrutinib might modify the cell membrane tension and elasticity through a mechanism that is worthy of further studies. These observations might also hint a role of ibrutinib in restoring the mechanoreciprocity of leukemic cells in the tissue microenvironment. To support this hypothesis, we undertook the first study of the mechanical properties of tissue-resident CLL cells isolated from the distinct BM and LN niches. Interestingly, a persistent stiffening of resident versus circulating cells was observed, indicating a possible involvement of ibrutinib in modulating the life cycle of CLL cells,^70^ by impairing the re-entry of CLL cells into tissues during drug treatment. This singular feature, then, might impact on a mechanism of resistance to ibrutinib,^11^ limiting CLL cells mobilization from the tissues where they possibly lurk during therapy. This hypothesis finds preliminary evidence on the results obtained on cells from a single CLL patient at the time of the ibrutinib resistance onset, for which the cellular mechanical properties reverted to those measured before the response to therapy.

Overall, our findings suggest that the pathological alteration of the intrinsic mechanical phenotype could be a possible mechanism of CLL cells retention within the tissue during progression and could be reverted by effective therapies. Detailed studies aimed at dissecting the relation between the mechanical properties of CLL cells and the involved signaling pathways are warranted in the future to define new potential therapeutic targets and strategies, aiming at the normalization of the mechanical fingerprints of the leukemic cells.

## ACKNOWLEDGMENTS

This study makes use of data generated by the Blueprint Consortium. A full list of the investigators who contributed to the generation of the data is available from https://www.blueprint-epigenome.eu. We thank Pamela Ranghetti, Eleonora Perotta, and Luca Russo for technical suggestions. We gratefully acknowledge Igor Sokolov for the helpful discussion.

## AUTHOR CONTRIBUTIONS

MS, VC, EB, FB, ADT, DB, CS, and RC performed the experiments. MS, EB, VC, DS, MZ, VLC, DB, OO, FM, CS, and RC analyzed the data. VC, VRC, MZ, PG, OO, FM, and CS supervised the activity. MS, EB, VC, CAM, FM, CS, and VRC wrote the article. LS and PG provided patients’ and clinical information. MS, EB, VC, DS, CAM, LS, PG, OO, VRC, MZ, FM, and CS revised the article.

## DATA AVAILABILITY

All data generated and analyzed during this study are included in this article and its supplemental digital content files.

## DISCLOSURES

OO is the cofounder of Zellmechanik Dresden commercialising real-time deformability cytometry. PG is a HemaSphere editor. All the other authors have no conflicts of interest to disclose.

## SOURCES OF FUNDING

CS project is supported by Associazione Italiana per la Ricerca sul Cancro AIRC under IG 2018 - ID 21332 project. OO gratefully acknowledges financial support from the German Federal Ministry of Education and Research (ZIK grant to OO under grant agreement no. 03Z22CN11) as well as from the German Center for Cardiovascular Research (Postdoc start-up grant to OO under grant agreement no. 81X3400107). CAM acknowledges financial support from the Italian Ministry of University and Research (MIUR) Department of Excellence project PREMIA (PREcision MedIcine Approach: bringing biomarker research to clinics). STED microscopy was conducted at the Microscopy & Dynamic Imaging Unit, CNIC, ICTS-ReDib, co-funded by MCIN/AEI/10.13039/501100011033, and FEDER “*Una manera de hacer Europa*” (#ICTS-2018-04-CNIC-16). The CNIC is supported by the Ministerio de Ciencia e Innovación and the Pro CNIC Foundation and is a Severo Ochoa Center of Excellence (CEX2020-001041-S). Schemes in figures 1, 2, 3 and 4 have been generated with BioRender.com. Funding for the project was provided by the European Union’s Seventh Framework Programme (FP7/2007-2013) under grant agreement no 282510 – BLUEPRINT.

## Supplementary Material


