# Supplementary material for: The Nanomechanical Properties of CLL Cells Are Linked to the Actin Cytoskeleton and Are a Potential Target of BTK Inhibitors
Source: Hemasphere. 2023 Jul 21;7(8):e931. doi: 10.1097/HS9.0000000000000931 (PMC10365208; doi:10.1097/HS9.0000000000000931)
Supplement: Supplementary file 1 [file hs9-7-e931-s001.docx]

Supplementary Information

# SUPPLEMENTARY METHODS

**Healthy samples**

# The buffy coats study was approved by the IRCCS Ospedale San Raffaele (OSR) ethics committee under the protocol Leu-Buffy coat entitled: “Characterization of leukocyte subpopulations from buffy coats”.

# Human primary sample purification

CD19 cells were negatively selected from fresh peripheral blood (PB), bone marrow (BM) and lymph nodes (LN) from patients or healthy donors using the RosetteSep B-lymphocyte enrichment kit (StemCell Technologies, [Vancouver, Canada](https://www.google.it/search?sxsrf=APwXEdep2cS8NnCq-2Ox4fl0t4QRutxWpQ:1682588877498&q=Vancouver&stick=H4sIAAAAAAAAAONgVuLQz9U3sDDIMHrEaMwt8PLHPWEprUlrTl5jVOHiCs7IL3fNK8ksqRQS42KDsnikuLjgmngWsXKGJeYl55eWpRYBAFVgH-xOAAAA&sa=X&ved=2ahUKEwjcieKC5Mn-AhXRS_EDHZtwA3EQzIcDKAB6BAgKEAE)). HD-B cells were further negatively selected using B-lymphocyte enrichment kit (StemCell Technologies, [Vancouver, Canada](https://www.google.it/search?sxsrf=APwXEdep2cS8NnCq-2Ox4fl0t4QRutxWpQ:1682588877498&q=Vancouver&stick=H4sIAAAAAAAAAONgVuLQz9U3sDDIMHrEaMwt8PLHPWEprUlrTl5jVOHiCs7IL3fNK8ksqRQS42KDsnikuLjgmngWsXKGJeYl55eWpRYBAFVgH-xOAAAA&sa=X&ved=2ahUKEwjcieKC5Mn-AhXRS_EDHZtwA3EQzIcDKAB6BAgKEAE)). The purity of all preparations was always higher than 99%, and the cells co-expressed CD19 and CD5 on their surface as assayed by flow cytometry (FC500; Beckman Coulter, Brea, California); preparations were virtually devoid of natural killer cells, T lymphocytes, and monocytes. After purification, cells were immediately frozen for further experiments.

# Immunofluorescence Staining

Primary cells were seeded on coated polyornithine (1:5) coverslip (22 × 22-mm high-precision glass, code: 0101050, Paul Marienfeld GmbH & Co. KG, Lauda-Königshofen, Germany) and incubated for 2 h at 37°C and 5% CO_2_. For single and double immunostaining, cells were washed with PBS and fixed with PFA 4%, incubated for 15 min in the dark at room temperature, and permeabilized in blocking buffer solution (blocking buffer: 0.1% w/v BSA, 10% v/v FBS in PBS), containing 0.3% v/v Triton-X 100 (Sigma- Aldrich, Merck, Germany), to limit nonspecific antibody binding. Samples were then incubated overnight at 4°C with primary monoclonal anti-mouse beta actin monoclonal (BA3R) antibody (code: MA5-15739, Thermo Fisher Scientific, [Waltham, Massachusetts,](https://www.google.it/search?sxsrf=APq-WBusg9zRrtK-OZ4LjB9z_G1lGu5T5Q:1646232430203&q=Waltham&stick=H4sIAAAAAAAAAOPgE-LUz9U3MDNLKUxS4gAxM6qMTbW0spOt9POL0hPzMqsSSzLz81A4VhmpiSmFpYlFJalFxYtY2cMTc0oyEnN3sDICANGzN1FQAAAA&sa=X&ved=2ahUKEwig7ODZ1af2AhVBQvEDHYScBA8QmxMoAXoECBwQAw) USA), primary monoclonal myosin light chain 2 Antibody (code: 3672S, Cell Signaling Technology, [Danvers, Massachusetts,](https://www.google.it/search?sxsrf=APq-WBvwDpRvXuKyT1G2s56Y7EVkEPnuXw:1646232372823&q=Danvers&stick=H4sIAAAAAAAAAOPgE-LSz9U3MCu3KKlIUeIEsQ1zKsrztLSyk63084vSE_MyqxJLMvPzUDhWGamJKYWliUUlqUXFi1jZXRLzyoCsHayMAAj2gl1SAAAA&sa=X&ved=2ahUKEwj-yLK-1af2AhXPasAKHZVSAcgQmxMoAXoECCwQAw) USA) or both for co-staining, then labeled with an goat-anti-mouse-Alexa568 (code: A-11031, Thermo Fisher Scientific, [Waltham, Massachusetts,](https://www.google.it/search?sxsrf=APq-WBusg9zRrtK-OZ4LjB9z_G1lGu5T5Q:1646232430203&q=Waltham&stick=H4sIAAAAAAAAAOPgE-LUz9U3MDNLKUxS4gAxM6qMTbW0spOt9POL0hPzMqsSSzLz81A4VhmpiSmFpYlFJalFxYtY2cMTc0oyEnN3sDICANGzN1FQAAAA&sa=X&ved=2ahUKEwig7ODZ1af2AhVBQvEDHYScBA8QmxMoAXoECBwQAw) USA), goat-anti-rabbit-Alexa532 (code: A-11009, Thermo Fisher Scientific, [Waltham, Massachusetts,](https://www.google.it/search?sxsrf=APq-WBusg9zRrtK-OZ4LjB9z_G1lGu5T5Q:1646232430203&q=Waltham&stick=H4sIAAAAAAAAAOPgE-LUz9U3MDNLKUxS4gAxM6qMTbW0spOt9POL0hPzMqsSSzLz81A4VhmpiSmFpYlFJalFxYtY2cMTc0oyEnN3sDICANGzN1FQAAAA&sa=X&ved=2ahUKEwig7ODZ1af2AhVBQvEDHYScBA8QmxMoAXoECBwQAw) USA) or both, for 2h at RT and in the dark. Samples were then washed with PBS and mounted with ProLong Gold antifade reagents (Invitrogen, Thermo Fisher Scientific, USA). For Ibrutinib treatment, cells were plated on pre-coated polyornithine 1:5 dishes for 1h in controlled condition. At the end of the incubation, 1uM Ibrutinib (Selleckchem, S2680, Planegg, Germany) was added directly in the dish and incubated for 2h at 37°C and 5% CO_2_. Cells were then fixed and permeabilized and double immunostaining was performed following the protocol described above.

**STED microscopy**

Cells stained for Alexa532 immunolabeled myosin or Alexa568 immunolabeled actin were imaged with X, Y depletion at 660 nm set at 80% laser power, with a gated unidirectional resonant scanning mode at 8,000 Hz. For actin filament analysis, excitation was at 578 nm by a white laser at 15% power. Images were acquired at 48-line and 6-frames average per optical section. For myosin quantification, excitation was at 525 nm by a white laser at 15% power. Images were acquired at 48-line and 9-frames average per optical section. Fluorescence (589 nm - 632 nm) for actin and (531 nm - 562 nm) for myosin was collected using a HyD spectral detector in standard mode and applying a gating of 0.2 and 0.3 ns respectively. A zoom of 3.5 times was applied to optical sections of 1248 x 1248 pixels acquired at the top, equatorial and bottom regions of the cells, with a pixel size of 33 x 33 nm.

To perform colocalization analysis of Alexa532 immunolabeled myosin and Alexa568 immunolabeled actin, samples were acquired with X, Y with depletion at 660 nm and 80% power for actin and 100% power for myosin, with a gated unidirectional scanning mode at 700 Hz. Fluorophores were excited at 583 nm (18% white laser power) and 520 nm (39% white laser power) for actin and myosin respectively. Fluorescence was collected using two HyD spectral detectors in standard mode with a gaiting of 0.3 ns. Spectral detection was set at 530 nm - 546 nm for myosin and 608 nm - 632 nm for actin. A zoom of 2 times was applied to collect optical sections of 3200 x 3200 pixels acquired at the bottom and equatorial sections of cells, with a pixel size of 58 x 58 nm. This setting was chosen among others to maximize the X, Y resolution, minimize photobleaching, avoid crosstalk between the two channels, autofluorescence and reflections, keeping the white laser excitation at the minimum, yet being capable of maintaining the same settings for all cells, which showed variable intensities. Depletion laser power, image format, zoom, and scanning conditions were optimized according to the same principle to obtain minimal photobleaching. The image resolution under all these conditions optimized for our samples was determined by measuring the X, Y PSF with a 23-nm nanobead sample coated with the Alexa568 and Alexa532 (GATTAquant GmbH, Gräfelfing, Germany) (**Fig. S4**).

**Image Analysis**

All images were analyzed by ImageJ/Fiji software^1^. STED images were post-processed for background subtraction with a rolling ball radius of 30 and deconvolved applying the CLME algorithm to each optical section (Huygens software, Scientific Volume Imaging BV, Hilversum, The Netherlands). Then images were cropped to define single cell ROIs. To determine the actin density, the cellular perimeter was set as reference ROI by thresholding (ImageJ/Fiji, Triangle algorithm). The actin inside the cellular ROI was segmented (ImageJ/Fiji Moment thresholding). We consider the total area as the occupation value of the intensity inside the reference ROI. The same method was used for myosin density analysis (**Fig. S1**). For measuring actin filament, images were post-processed for background subtraction, followed by deconvolution, and contrast enhancement (ImageJ/Fiji, CLAHE function). Actin distribution was then detected by applying the ImageJ/FiJi LoG3 PlugIn^2^. Finally, images were converted into binary masks (ImageJ/Fiji, Phansalkar auto local threshold algorithm, radius of 20) and skeletonized for measuring the length of actin filaments and branches (**Fig. S3**). For statistical analysis filaments shorter than 260 nm were discarded. For actomyosin colocalization, after deconvolution optical sections were single cell cropped to minimize the background around each cell. Colocalization analysis was performed using the JACoP plugin in ImageJ/Fiji^3^ applying the Moment thresholding to both channels (**Fig. S6**). We considered the Manders’ coefficient 2 (Channel 2: actin on Channel 1: myosin). We also performed the Costes’ randomized test^3^ to prove the no-randomness of the results.

# AFM-FS data analysis

## The mechanical properties of a complex system such as a cell were described through several theoretical models returning different values of the mechanical parameters^4,5^. The most common model describing the elastic properties of cells is the Hertz model^6,7,8^, corrected by Sneddon considering the tip geometry^9^ that affects the contact area. Following this model, the relation between the loading force *F*, the Young’s Modulus *E*, and the resulting indentation $\delta$ for a pyramidal tip is:

## $F=E' tan(\alpha) \delta^{2}/\surd2$

## Where $\alpha$ is the face tip angle and $E'=E/(1-\mu^{2})$ is the reduced Young’s Modulus that takes into account the cell Poisson ratio $\mu$, usually assumed to be 0.5. As shown in **Fig. 3D**, the acquired data are well described by the Hertz-Sneddon model. It has been observed that this is not always the case and deviations from the Hertz-Sneddon model have been often reported^10^. We faced this issue with an operational approach by selecting all the curves which were more effectively described by the model and disregarding the others. It is also worth noting that the absolute value of Young’s Modulus is difficult to determine. For this reason, no matter what model is applied, it is useful to use a differential approach in which all results are compared to a reference sample. Here we used as reference sample B lymphocytes from healthy donors and cells treated with drugs of known effect.

## Tips with different shapes are available on the market for AFM-FS measurements, and recently a lot of authors choose the colloidal probes for the investigation of cell stiffness^11^. In this work, we performed stiffness measurements with pyramidal probes in order to have an easier experimental strategy. Indeed, several attempts to acquire reproducible force-indentation curves on B lymphocytes with colloidal probes (Novascan Technologies, Chicago, USA, on 0.06 N/m cantilevers) or tipless cantilever (Bruker, MLCT-O10, [Billerica, Massachusetts,](https://www.google.it/search?sxsrf=APwXEdePHeZ2fjlr8gBdk8HvPXhWll_bKg:1682589226215&q=Billerica&stick=H4sIAAAAAAAAAONgVuLQz9U3KKmqynjEaMwt8PLHPWEprUlrTl5jVOHiCs7IL3fNK8ksqRQS42KDsnikuLjgmngWsXI6ZebkpBZlJicCADaURGhOAAAA&sa=X&ved=2ahUKEwjKnIap5cn-AhW2X_EDHdnWCk4QzIcDKAB6BAghEAE) USA) resulted in buckling contact points and sliding cells (data not shown). In addition, the use of pyramidal probes allows us to measure the cellular cortical stiffness, since the small tip and the reduced indentation allows probing the cortex mechanical properties^129^.

**RT-DC data analysis**

RT-DC measures the deformation *D* of a cell, which is defined:

$D=1-C=1-2\frac{\sqrt{\pi A}}{P}$

where *C* is its circularity, *A* the area and *P* the perimeter. For an ideal circle, *D* = 0, and for any deformation at constant volume $D>0$^13^. The analysis is performed in real-time on thousands of cells in a single experimental run and an analytical as well as numerical model enables derivation of the Young's Modulus^14,15^.

# Viability counts for AFM-FS experiments

## To exclude that cell death could affect the experiments, a control petri dish was kept in the incubator in the same conditions of the cells under measure. Cells were counted by trypan blue staining. No significant differences were found in terms of cell viability at the end of the measurement window (maximum 2h, **Fig. S11**). However, beyond this temporal window the viability of both HD-B and CLL cells started to decrease steeply, drastically reducing the number of possible measurements for every patient.

**Protein lysis and Western Blot (WB)**

Cells were lysed on ice for 15 minutes in RIPA Buffer (Sigma-Aldrich, Burlington, Massachusetts, USA) with fresh protease and phosphatase inhibitors cocktail (Roche, [Basel](http://en.wikipedia.org/wiki/Basel), ‎[Switzerland](http://en.wikipedia.org/wiki/Switzerland)). Cells were then centrifuged at 13.000 rpm for 15 minutes at 4°C, and supernatants were collected and stored at −80°C until further use. Protein content was determined using the BCA protein assay kit (Bio-Rad, Hercules California USA) according to the manufacturer’s instructions. The total of protein content of about 5x106 cells was supplemented with NuPage Sample Buffer (4x) and NuPage Sample Reducing Agent (10x) and loaded onto 4–12% sodium dodecyl sulfate-polyacrylamide gradient gels (Invitrogen, [Waltham, Massachusetts,](http://en.wikipedia.org/wiki/Waltham,_Massachusetts) USA), then transferred to nitrocellulose membranes (Thermo Scientific, Waltham, Massachusetts, USA). Membranes were blocked for 1.30h in PBS-Tween containing 5% BSA and incubated overnight with the following primary antibodies: Anti-phospho myosin light chain II (cell signaling-3674s-Rabbit 1:1000) and Anti-myosin light chain II (Cell Signaling-3672s- Rabbit 1:1000, Danvers, Massachusetts, USA). Incubation with primary antibodies was followed by species-specific Horseradish Peroxidase (HRP)-conjugated secondary antibodies (anti-Rabbit-A16023-Invitrogen, [Waltham, Massachusetts,](http://en.wikipedia.org/wiki/Waltham,_Massachusetts) USA, diluted 1:10000) for 1h. All WB were normalized to anti β-actin HRP conjugated (Cell Signaling, Rabbit, Danvers, Massachusetts, USA, 1:50000). Amersham ECL Western Blotting Analysis System from GE Healthcare was used to visualize immuno-reactive bands. Western blots were acquired using Biorad Chemidoc (Bio Rad, Hercules, California, USA) and quantification of relative protein expression levels was performed using Image Lab Software for PC Version 6.1. Graphs and statistical analysis were performed using GraphPad Prism (San Diego, California, USA, https://www.graphpad.com/scientific-software/prism/).

#

#

#

# SUPPLEMENTARY FIGURES


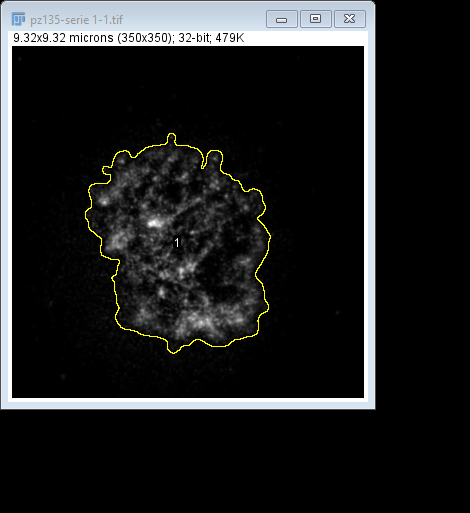


**A**


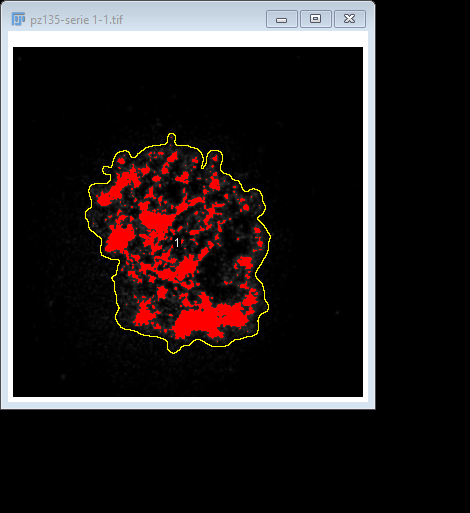


**B**

**Fig. S1 Example of analysis of actin or myosin density in 2D-STED optical sections.**

**(A)** Images were post-processed for background subtraction, followed by deconvolution, and selection of the cell total area (yellow trace ROI). **(B)** The ImageJ/FiJi threshold Moment algorithm was applied to select the fluorescent fraction inside the cell ROI and obtain the density of myosin in the single cell


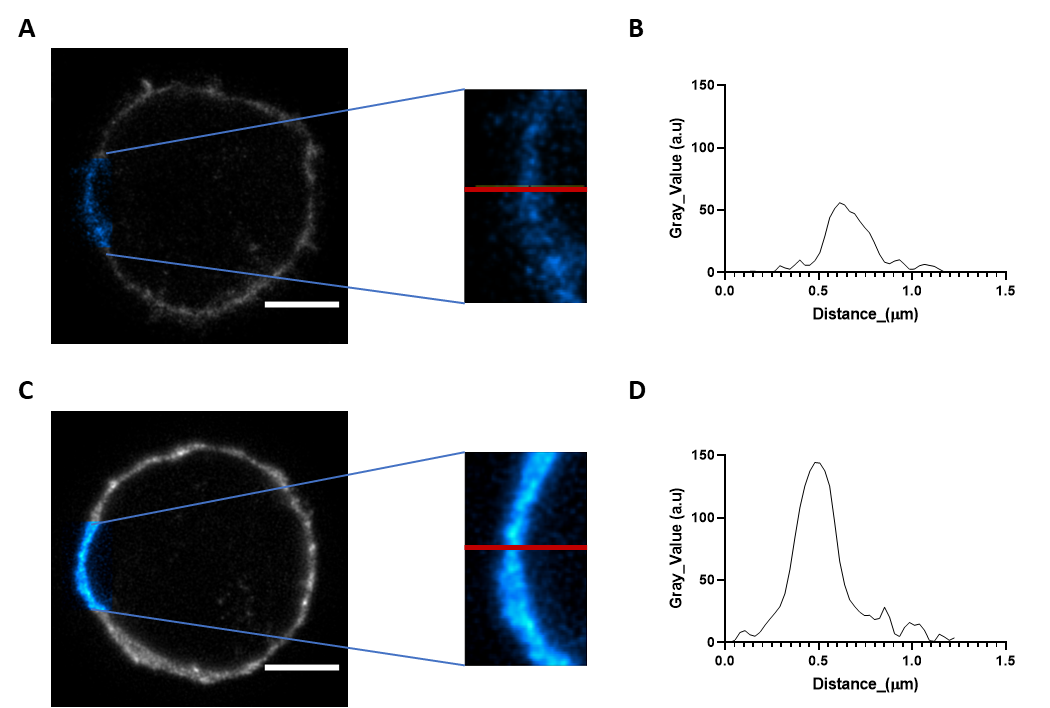


**Fig. S2 Example of actin density in 2D-STED equatorial optical section in single cell.**

Representative HD-B **(A)** and CLL **(C)** cells and corresponding intensity profiles **(B, D)** drawn by a central line (red) in a zoomed ROI. Scale bar = 2µm.


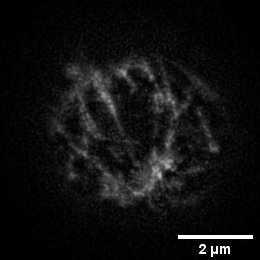


**A**


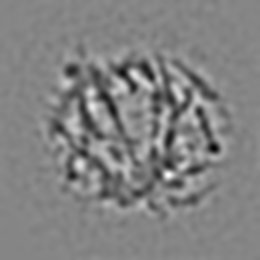


**B**


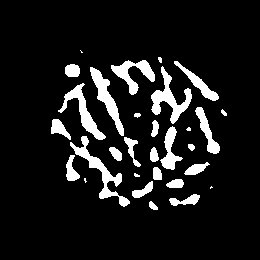


**C**


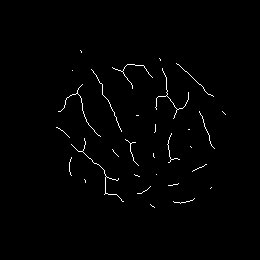


**D**

**Fig. S3 Steps for actin filament length and branching analysis in 2D-STED optical sections.**

**(A)** Images were post-processed for background subtraction, followed by deconvolution, and contrast enhancement; **(B)** actin distribution was then detected by applying the ImageJ/FiJi LoG3 PlugIn, **(C)** finally images were converted into binary mask **(D)**, and skeletonized for measuring the length of actin filaments and branches by ImageJ/FiJi.


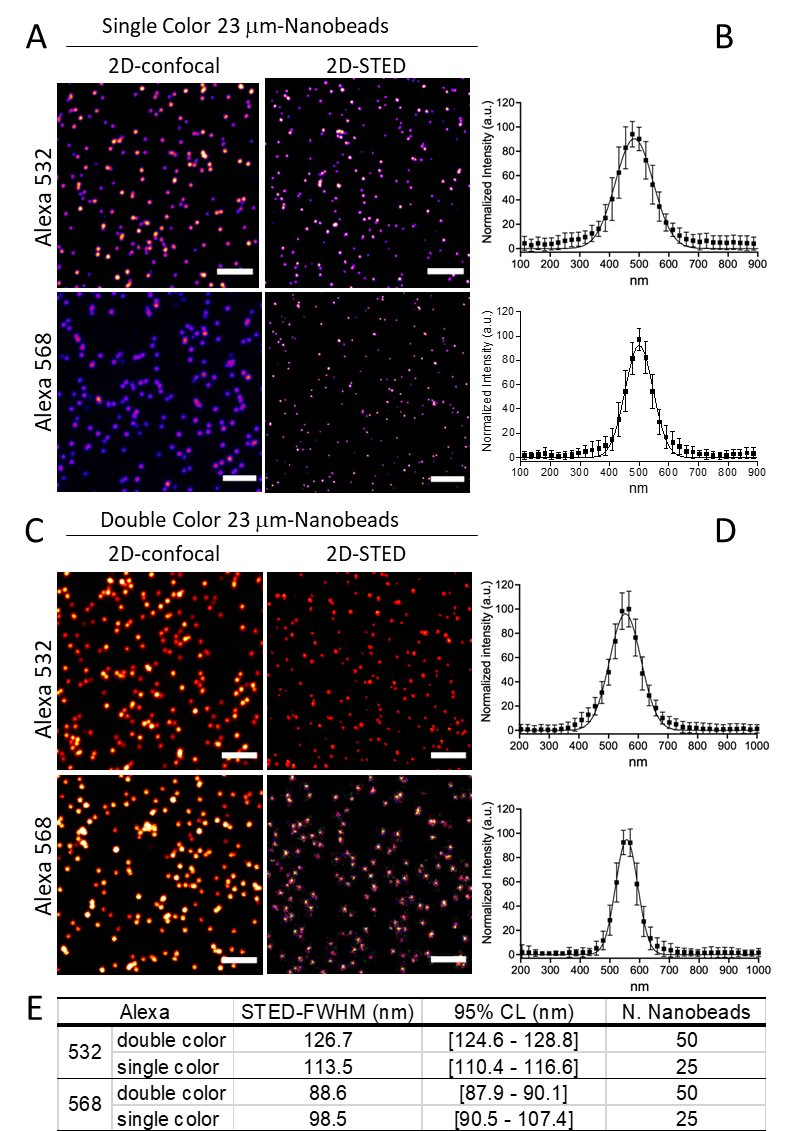


**Fig. S4 Single and Double color 2D-STED optical resolution.**

The optical resolution in 2D-STED anti-myosin-Alexa532 and anti-actin-Alexa569 images was determined by measuring the FWHM of fluorescent 23 mm nanobeads depleted at the experimental conditions applied to either to single or double-stained cells with 660 nm STED depletion: Representative confocal and STED images of single color **(A)** and double color **(C)** Alexa-nanobeads. Gaussian fits +/- SD of nanobead normalized fluorescence intensities X,Y-profiles averaged on replicate measurements are shown in **(B)** and **(D)**. FWHM values obtained for single- and double-stained nanobeads are shown in **(E)**. Scale bar = 2 𝜇m.


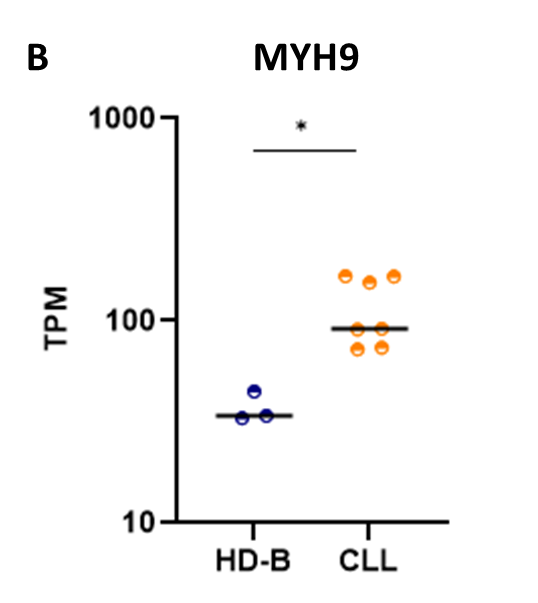

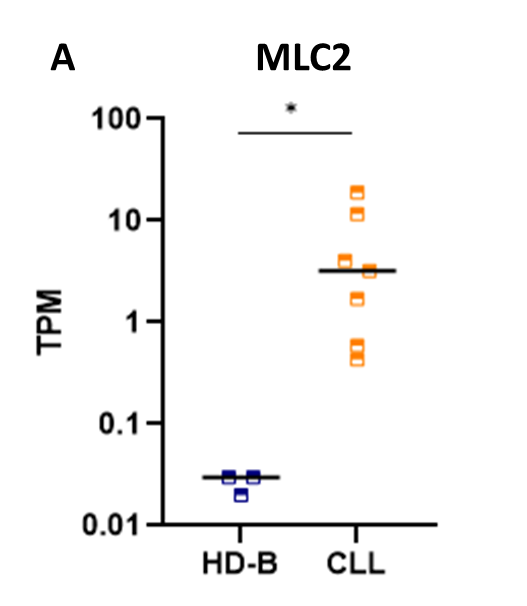


**Fig. S5 Myosin expression.**

*In-silico* analysis (Blueprint Consortium) on primary HD-B and CLL cells isolated from peripheral blood of the protein level expressed as Transcripts Per Million (TPM) of **(A)** myosin regulatory light chain 2 (MLC2, p value = 0.016) and **(B)** myosin heavy chain, non-muscle IIa (MYH9, p value = 0.017).


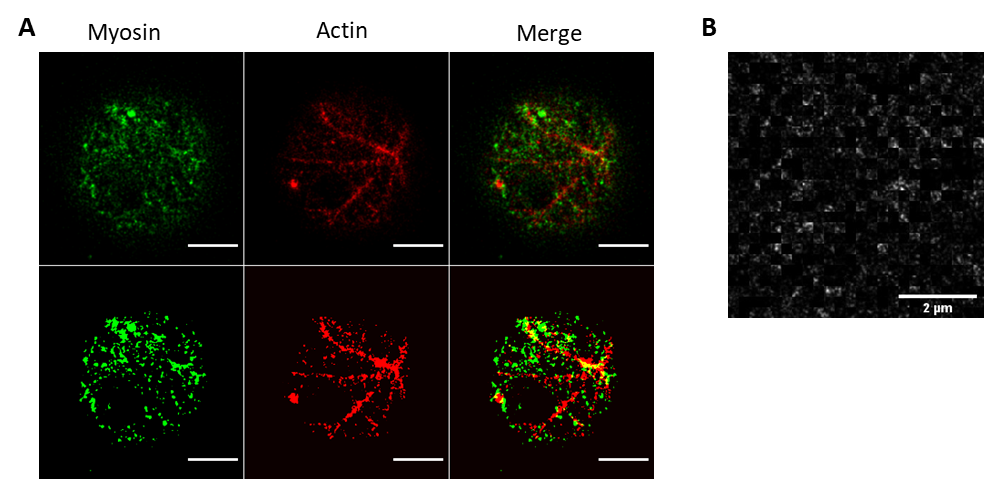


**Fig. S6 Representative example of colocalization analysis of double color 2D-STED optical sections.**

**(A)** Anti-myosin-Alexa532, anti-actin-Alexa568 and merged images of bottom regions in a representative CLL cell (top), and the corresponding binary masks obtained after thresholding (bottom) that were used for computing the colocalization score by JACoP plug-In in ImageJ/Fiji **(B)** Costes’ Randomization control test. Scale bars = 2µm.


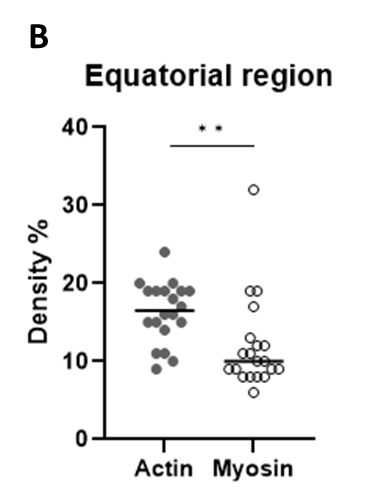

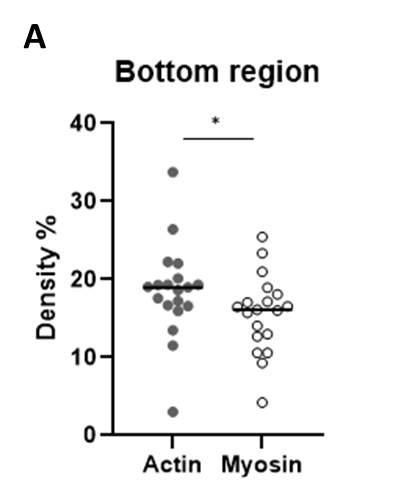


**Fig. S7 Actin and myosin densities in co-stained 2D-STED images.**

**(A)** Density % of actin *vs* myosin on bottom cellular sections. Actin median 19.0 (CI 16.6/20.1) and myosin median 16.1 (CI 12.7/18.1) (p value = 0.03). **(B)** Density % of actin *vs* myosin in equatorial cellular sections actin median 16.5 (CI 15.0/19.0) and myosin median 10.0 (CI 9.0/12.0) (p value = 0.001). HD-B are represented in dark gray and CLL are represented in light gray. (19 images were analyzed for both the 2 sections). In each optical section actin density has a higher median compared to myosin.


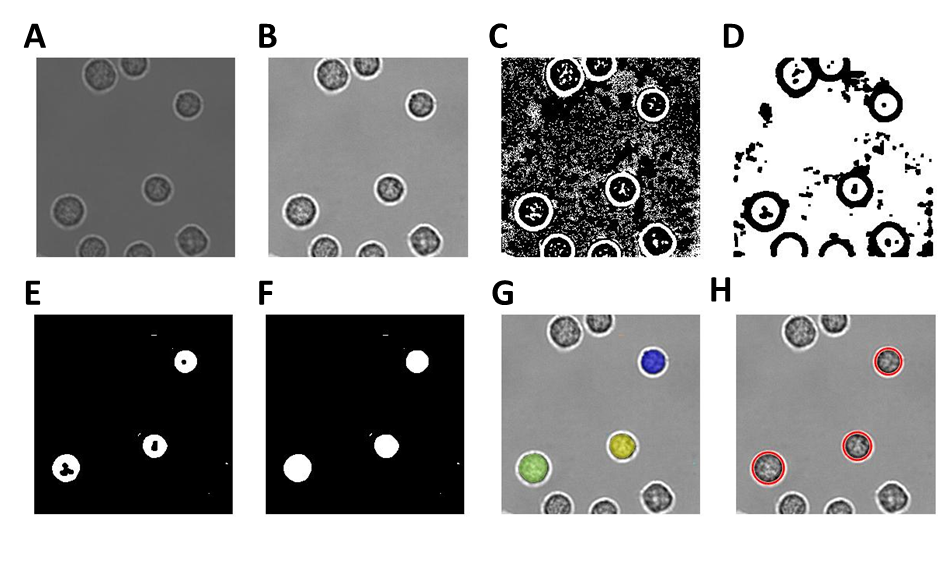


**Fig. S8 Cell segmentation procedure for swelling experiments.**

**(A)** Original Image. **(B)** Intensity equalized image. **(C)** H-maxima transformation image. **(D)** Inverted image. **(E)** Cleared border Image. **(F)** Convex transformation image. **(G)** Labeled object superimposed to the equalized image. **(H)** Circles of maxima diameter superimposed to the equalized image.

**Fig. S9 Example of a time-radius cell growing during hypoosmotic stress response**.
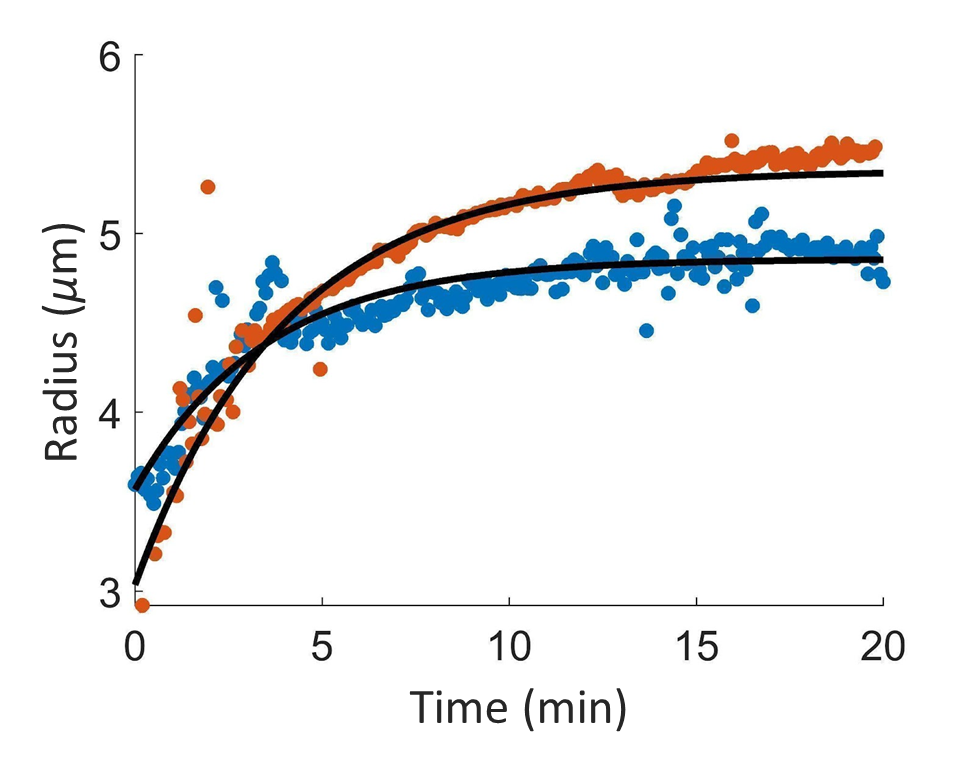


Dots are the extracted radius for each timepoint (blue for HD-B cell and orange for CLL cell) using the segmentation procedure described in material and methods, the continuous black lines represent a fit to the data with an exponential increasing function ((R_fin_-R_in_)*(1-exp(-t/τ))+R_in_) where t is the time and the free parameters are the final and initial radius R_fin_ and R_in_ and the characteristic growing time τ.


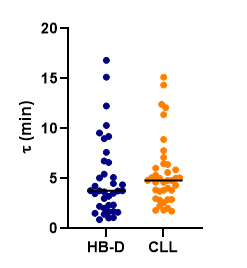


**Fig. S10 Distribution of τ values** from swelling kinetics, obtained from fitted exponentially increasing functions for HD-B and CLL cells. HD-B median 3.8 min, and CLL median 4.8 min, (p value = 0.1). 37 HD-B cells from 3 donors and 39 CLL cells from 4 patients.

**Fig. S11 Control of cell viability during AFM-FS experiments.**
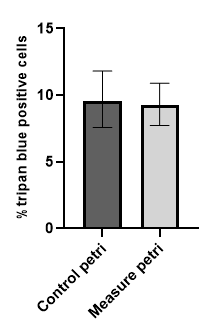


Histogram of the percentage of dead cells in adhesion on a poly-L-ornithine coating in PBS (both CLL (n=1) and HD-B (n=1)) for the petri dish before (dark gray) and after (light gray) AFM-FS measurement showing that AFM-FS measurements do not affect cell viability. Control means 9.4 (CI 7.5/11.6) and measured mean 9.1 (CI 7.8/10.7).

##
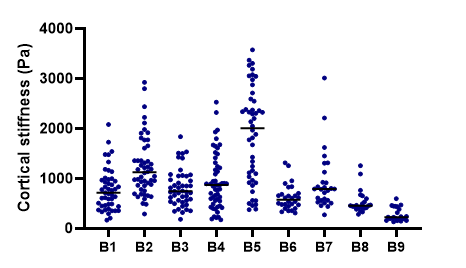


**Fig. S12 Distribution of cortical stiffness in cells from single HD-B donors.**

In the scatter plot, each dot represents the median cortical stiffness of each single cell measured during the AFM-FS analysis for each HD-B donor (7). The bar shows the median for each single sample. B1 = 47 cells, B2 = 51 cells, B3 = 45 cells, B4 = 54 cells, B5 = 50 cells, B6 = 28 cells, B7 = 27 cells, B8 = 23 cells, B9 = 23 cells.

**Fig. S13 Distribution of single CLL patients' cortical stiffness.**
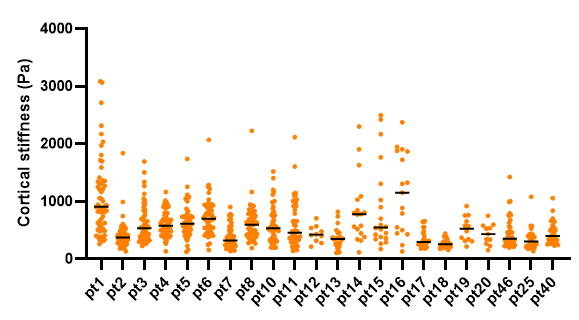


In the scatter plot each dot corresponds to the median cortical stiffness of each single cell measured during the AFM-FS analysis for each CLL patient (15). The bar shows the median for each single sample. Pt-1 = 58 cells, pt-2 = 45 cells, pt-3 = 50 cells, pt-4 = 52 cells, pt-5 = 42 cells, pt-6 = 53 cells, pt-7 = 52 cells, pt-8 = 52 cells, pt-10 = 51 cells, pt-11 = 52 cells, pt-12 = 10 cells, pt-13 = 24 cells, pt-14 = 18 cells, pt-15 = 20 cells, pt-16 = 17 cells, pt-17 = 26 cells, pt-18 = 18 cells, pt-19 = 12 cells, pt-20 = 11 cells, pt- 46 = 41 cells, pt-25 = 31 cells, pt-40 = 30 cells.

**
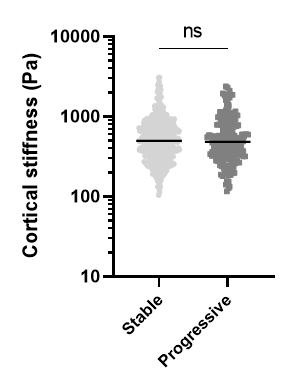
**

**B**

**A**

**Fig. S14 Cortical stiffness of CLL patients used in for the analysis classified based on clinical and biological prognostic markers (Table I).**
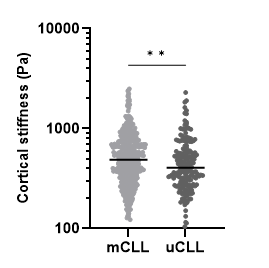


**(A)** Scatter plot of CLL cortical stiffness classified based on the mutational status (patients mCLL n=16, uCLL n=5) of the IGHV chain (p value = 0.04). **(B)** Scatter plot of CLL cortical stiffness based on the clinical course of the disease (p value = 0.7). Patients Stable n=16, progressive n=6.


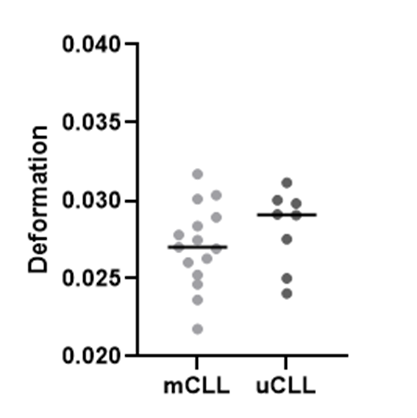

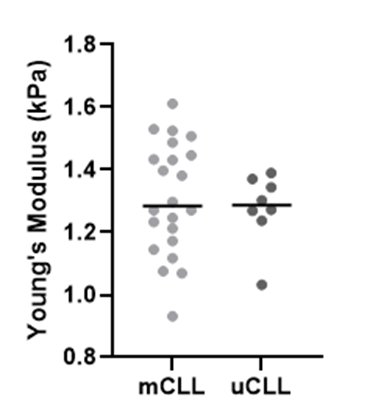

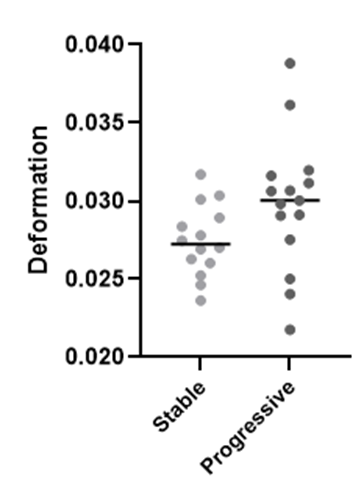

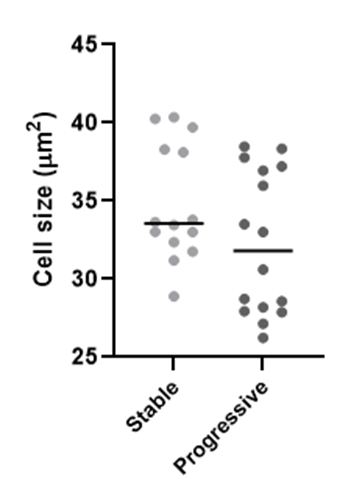

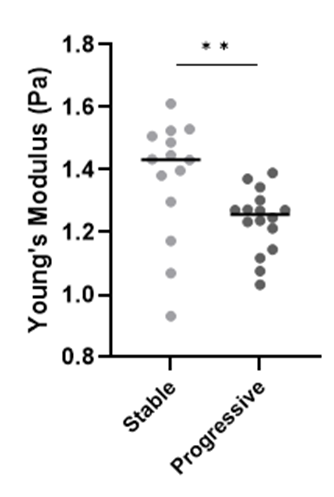

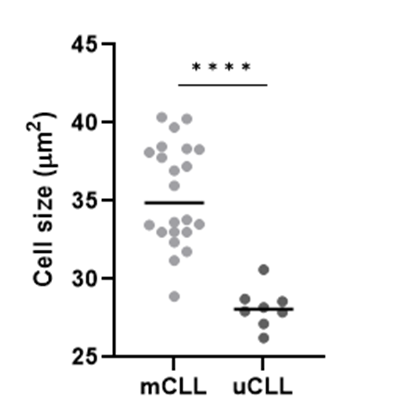


**Fig. S15 RT-DC parameters based on diagnostic markers (Table1).**

**(Upper panel)** Scatter plots of cellular deformation (p value = 0.3), Young’s Modulus (p value = 0.7) and cellular area (p value < 0.0001) respectively of CLL cells based on the mutational state of the IGHV chain. In light gray mutated patients for the IGHV chain (mCLL= 8), in dark gray unmutated patients for the IGHV chain (uCLL=3). **(Lower panel)** Scatter plots of cellular deformation (p value = 0.06), Young’s Modulus (p value = 0.007) and cellular size (p value = 0.09) respectively, of CLL cells classified based on the disease progression (stable=6 in light gray, progressive=5 in dark gray).

# Fig. S16 Cell viability upon treatment with specific stimuli. The histogram shows the percentage of dead cells in untreated samples (basal, black), in samples treated with ibrutinib for 4 hours (dark gray) and in samples treated with cytochalasin D for 2 hours (light gray).
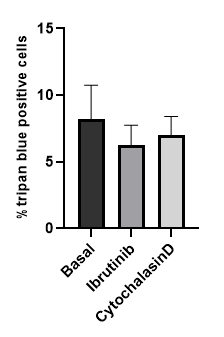


**Fig. S17 Cytochalasin D treatment.** Distribution of the cortical stiffness obtained by AFM-FS for HD-B and CLL cells before (unt= untreated) and after cytochalasin D treatment (+cytoD), p value < 0.0001.
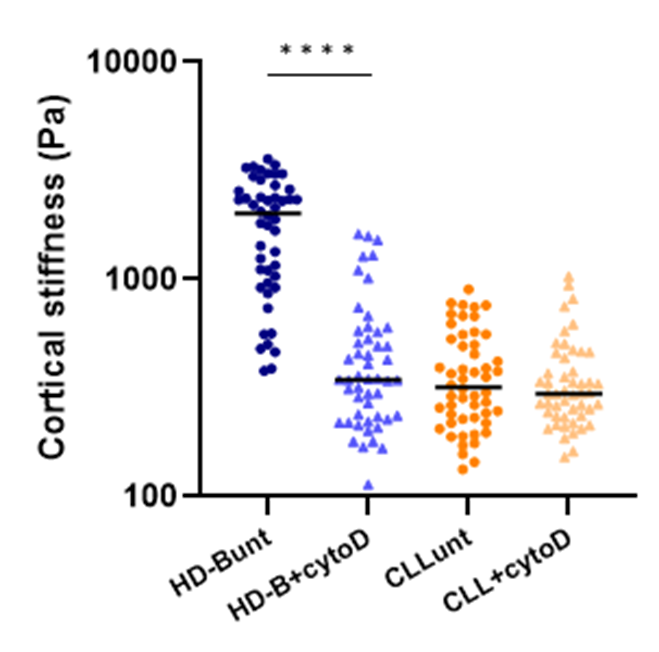


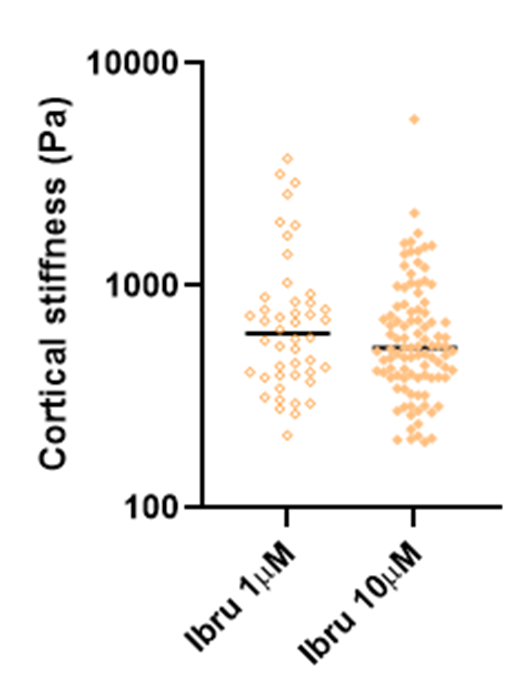


**Fig. S18 Ibrutinib treatment at different doses.** The cortical stiffness of CLL cells treated *in vitro* with Ibrutinib at 1µM and 10µM at the same incubation time, did not show significant difference (p value = 0.3), CLL patients n=3. Ibrutinib 1µM median 610.1 Pa (CI: 435/754 Pa) and ibrutinib 10µM median 526.7 Pa (CI: 476/591 Pa).


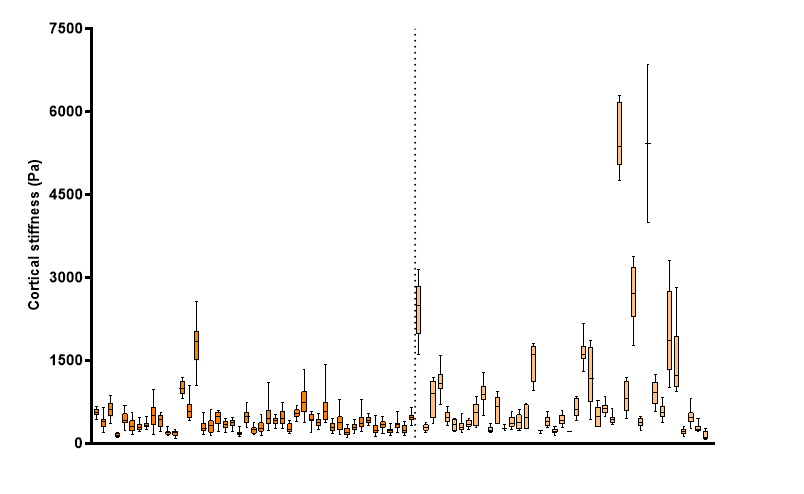


**Fig. S19 Representative whisker plot of single cell cortical stiffness** obtained by AFM-FS on cells from patients before and after *in vitro* treatment with ibrutinib for 4h. Each box plot represents a single cell, the bar represents the min and max values. Dark orange box plot represents untreated cells, light orange box plot represents treated patients with ibrutinib.

**
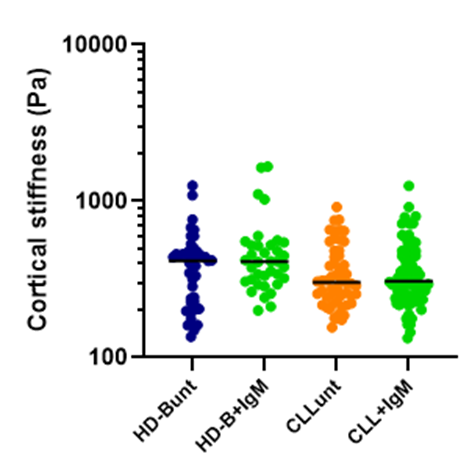
**

**Fig. S20 IgM stimulation.**  Scatter plot of the cortical stiffness by AFM of primary HD-B and CLL cells before (unt = untreated) and after (+IgM) stimulation with Anti-IgM. HD-Bunt median 416 Pa, (CI 284/450), HD-B+IgM, median 410 Pa (CI 333/480 Pa), p value = 0.3; CLLunt median 303 Pa (CI 261/341 Pa) and CLL+IgM median 307 Pa (CI 289/352 Pa), p value = 0.6. HD-Bunt n= 46 cells, HD-B+IgM n= 36 cells. from 2 donors. CLLunt n = 56 cells, CLL+IgM n= 86 cells, from 3 patients.


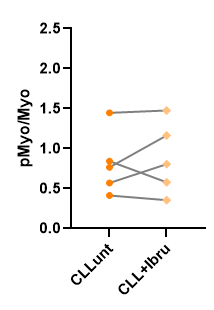

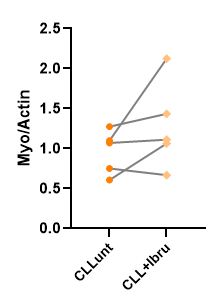


**B**

**A**

**Fig. S21 Western Blot analysis of CLL primary cells following ibrutinib treatment *in vitro*.**
Data were obtained from WB of primary CLL cells (n=5) before (CLLunt) and after 4h treatment with ibrutinib 10µM (CLL+Ibru). **(A)** Quantification of the myosin increases upon treatment with ibrutinib in single patients. The intensity of the myosin band was normalized as the ratio between myosin total protein and actin (housekeeping gene) bands **(B)** Quantification of the p-myosin increase upon treatment with Ibrutinib in single patients. p-myosin increase was normalized on the total myosin protein.
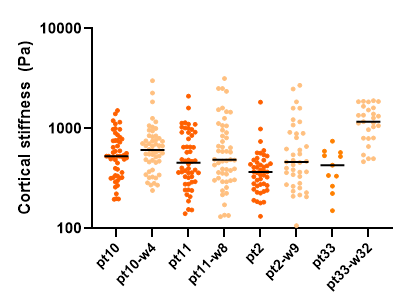


**Fig. S22 *In vivo* ibrutinib treatment.** Scatter plot showing the cortical stiffness in cells from single patients at the basal status and during treatment. Cells were collected from patient at different treatment times, week 4, 8, 9 and 32 respectively. The percentage of CD19+ CD5+ cells was at least 98% in all the samples analyzed.

**Fig. S23. Representative whisker plot of single cell cortical stiffness.** Data obtained by AFM-FS on cells from CLL patients *under ibrutinib clinical treatment*. Each box plot represents a single cell, the bar show the min and max values. Dark orange box plot represents cells of untreated patients, light orange bars represent patients treated with Ibrutinib.
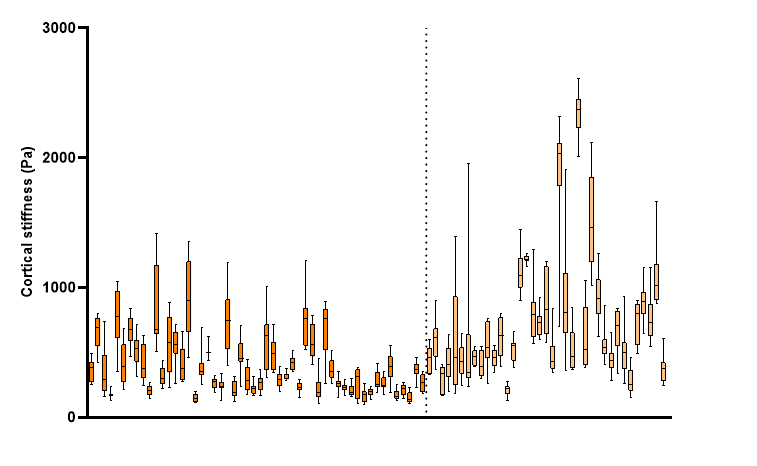


# LEGEND VIDEO

**Video S1 and S2. STED-3D rendering of CLL cells and HD-B cells stained for Alexa568 immunolabeled actin.** For the Z-stack we used a STED-3X-WLL SP8 microscope with a 100x/1.40 oil objective. Samples were acquired with X, Y, Z depletion at 660 nm and at 80% power in gated bidirectional resonant scanning mode at 8,000 Hz scan speed. Excitation was performed at 578 nm by a white laser at 15% power, acquiring 32-line average and 4-frames per optical section. Fluorescence (589 nm - 632 nm) was collected using a HyD spectral detector in standard mode and applying a gating of 0.2, zoom of 3 times, image format of 1248 x 659 x 106 pixels, with a voxel size of 31 nm x 31 nm x 80 nm. Images were deconvoluted with GLME algorithm (Huygens software, Scientific Volume Imaging BV, Hilversum, The Netherlands) and 3D rendering performed by Imaris software (Oxford Instruments).

**Video S3. Timelapse acquisition during swelling.** *Left panel:* Bright field time-lapse record of a single cell swelling kinetics after hypoosmotic shock. *Right panel:* Segmentation of single cell area during hypoosmotic stress.

**BIBLIOGRAPHY**

1. Schindelin J, Arganda-Carreras I, Frise E, et al. Fiji: an open-source platform for biological-image analysis. *Nat Methods 2012 97*. 2012;9(7):676-682. doi:10.1038/nmeth.2019

2. Sage D, Neumann FR, Hediger F, Gasser SM, Unser M. Automatic tracking of individual fluorescence particles: Application to the study of chromosome dynamics. *IEEE Trans Image Process*. 2005;14(9):1372-1383. doi:10.1109/TIP.2005.852787

3. Bolte S, Cordelières FP. A guided tour into subcellular colocalization analysis in light microscopy. *J Microsc*. 2006;224(3):213-232. doi:10.1111/j.1365-2818.2006.01706.x

4. Sokolov I, Dokukin ME, Guz N V. Method for quantitative measurements of the elastic modulus of biological cells in AFM indentation experiments. *Methods*. 2013;60(2):202-213. doi:10.1016/j.ymeth.2013.03.037

5. Dokukin ME, Sokolov I. On the measurements of rigidity modulus of soft materials in nanoindentation experiments at small depth. *Macromolecules*. 2012;45(10):4277-4288. doi:10.1021/ma202600b

6. Lekka M, Pabijan J. Measuring Elastic Properties of Single Cancer Cells by AFM. *Methods Mol Biol*. 2019;1886:315-324. doi:10.1007/978-1-4939-8894-5_18

7. Lekka M, Laidler P, Gil D, Lekki J, Stachura Z, Hrynkiewicz AZ. Elasticity of normal and cancerous human bladder cells studied by scanning force microscopy. *Eur Biophys J*. 1999;28(4):312-316. doi:10.1007/S002490050213

8. Lekka M. Discrimination Between Normal and Cancerous Cells Using AFM. *Bionanoscience*. 2016;6(1):65-80. doi:10.1007/S12668-016-0191-3

9. Sneddon IN. The relation between load and penetration in the axisymmetric boussinesq problem for a punch of arbitrary profile. *Int J Eng Sci*. 1965;3(1):47-57. doi:10.1016/0020-7225(65)90019-4

10. Guz N, Dokukin M, Kalaparthi V, Sokolov I. If cell mechanics can be described by elastic modulus: study of different models and probes used in indentation experiments. *Biophys J*. 2014;107(3):564-575. doi:10.1016/J.BPJ.2014.06.033

11. Iyer S, Gaikwad RM, Subba-Rao V, Woodworth CD, Sokolov I. Atomic force microscopy detects differences in the surface brush of normal and cancerous cells. *Nat Nanotechnol*. 2009;4(6):389-393. doi:10.1038/nnano.2009.77

12. Lei K, Kurum A, Kaynak M, et al. Cancer-cell stiffening via cholesterol depletion enhances adoptive T-cell immunotherapy. *Nat Biomed Eng*. 2021;5(12):1411-1425. doi:10.1038/s41551-021-00826-6

13. Otto O, Rosendahl P, Mietke A, et al. Real-time deformability cytometry: On-the-fly cell mechanical phenotyping. *Nat Methods*. 2015;12(3):199-202. doi:10.1038/nmeth.3281

14. Herbig M, Kräter M, Plak K, Müller P, Guck J, Otto O. Real-Time Deformability Cytometry: Label-Free Functional Characterization of Cells. *Methods Mol Biol*. 2018;1678:347-369. doi:10.1007/978-1-4939-7346-0_15

15. Mokbel M, Mokbel D, Mietke A, et al. Numerical Simulation of Real-Time Deformability Cytometry To Extract Cell Mechanical Properties. ACS Biomater Sci Eng.2017;3:2962-2973. doi:10.1021/acsbiomaterials.6b00558
